# Biodiversity Buffers Phytoplankton Biomass Production Against Nanoparticle Pollution Through Increased Facilitation

**DOI:** 10.1111/ele.70253

**Published:** 2025-11-09

**Authors:** Yiping Zuo, Qianna Xu, Michael Southard, Guangxing Zhang, Lan Gan, Hao Zhang, Pu Yan, Louis Walls, Mary Steinbeck, Muhan Zhao, Yongsheng Chen, Lin Jiang

**Affiliations:** ^1^ School of Biological Sciences Georgia Institute of Technology Atlanta Georgia USA; ^2^ The Woodruff School of Mechanical Engineering Georgia Institute of Technology Atlanta Georgia USA; ^3^ School of Civil and Environmental Engineering Georgia Institute of Technology Atlanta Georgia USA

**Keywords:** biodiversity, complementarity effect, facilitation, nanoparticles, phytoplankton, selection effect

## Abstract

Although biodiversity is widely recognised for its role in maintaining ecosystem functioning under environmental change, its importance has received relatively little attention in the context of emerging environmental stressors such as nanoparticles. By assembling phytoplankton communities across a gradient of species richness and exposing them to varying concentrations of copper oxide nanoparticles, we experimentally explored how biodiversity modulates ecosystem functioning under nanoparticle exposure. We found that the positive effect of biodiversity on phytoplankton biomass production was amplified at higher nanoparticle concentrations, allowing diverse communities to maintain biomass despite strong inhibitory effects of nanoparticles on monocultures. This enhanced biodiversity effect was primarily driven by increased complementarity, specifically through more frequent facilitative interactions among species under nanoparticle stress. Our findings advance the understanding of how biodiversity preserves ecosystem functioning and underscore its role in mitigating the impacts of emerging anthropogenic stressors.

## Graphical abstract

Biodiversity can buffer ecosystems against environmental stressors, yet its role under nanoparticle pollution remains unclear. Using freshwater phytoplankton communities exposed to copper oxide nanoparticles, we show that higher species richness alleviates productivity losses through increased facilitation among species. These findings highlight biodiversity's importance in sustaining ecosystem functioning under emerging contaminants.

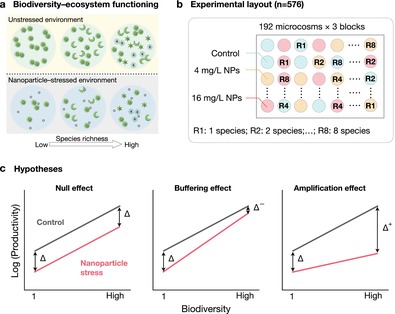

## 1 Introduction

Biodiversity has been a central focus of ecological research for the past three decades (Hector et al. 1999; Isbell et al. 2023; Mi et al. 2021; Tilman et al. 1996, 2014), not merely for its aesthetic value, but because of its critical role in maintaining ecosystem functions (Cardinale et al. 2006; Hooper et al. 2005; Loreau et al. 2001; Tilman et al. 2014). Among numerous lines of evidence, the most widely documented is the positive relationship between primary producer diversity and productivity across both terrestrial and aquatic ecosystems (Cardinale et al. 2007, 2011; Chen et al. 2025). In addition to the well‐established biodiversity effects under stable or ambient conditions, the accelerating pace of global change in the Anthropocene has further elevated the ecological significance of biodiversity, particularly its potential to buffer fluctuations in ecosystem functioning under environmental change (Oliver et al. 2015; Xu et al. 2021; Yachi and Loreau 1999). Beyond separately impacting biodiversity and ecosystem processes, environmental change can also influence the biodiversity–ecosystem functioning (BEF) relationship by either amplifying or dampening the biodiversity effects (Bestion et al. 2021; García et al. 2018; Hautier et al. 2014; He et al. 2022; Hong et al. 2022; Wang et al. 2023). Except under extreme stress conditions that exceed species' tolerance and may collapse positive biodiversity effects (Baert et al. 2018; Shovon et al. 2024), the general pattern is that positive biodiversity effects tend to be stronger under adverse environmental conditions that inhibit ecosystem functioning (García et al. 2018; Hong et al. 2022; Steudel et al. 2012), thereby buffering against negative impacts. However, most existing experiments manipulating biodiversity and environmental conditions have focused on global change drivers such as climate change and nutrient (e.g., nitrogen) enrichment (Cheng et al. 2024; Hong et al. 2022), with only a few addressing traditional chemical pollutants such as heavy metals or herbicides (Baert et al. 2016; Li et al. 2010). In contrast, emerging chemical pollutants are increasing at a much faster pace than climate change or traditional pollutants due to accelerating global chemical production (United Nations Environment Programme 2019), yet they have received far less attention in biodiversity research. In particular, the role of biodiversity in mediating ecosystem responses to nanoparticles remains largely unexplored.

Nanoparticles, defined as materials with at least one dimension between 1 and 100 nm, possess unique physicochemical properties distinct from their bulk counterparts (Jeevanandam et al. 2018; Roduner 2006). Their increasing use across industries (Janković and Plata 2019; Khan et al. 2025) has led to growing environmental release throughout the product life cycle—including manufacturing, use and disposal—with a significant portion entering aquatic ecosystems via runoff (Keller et al. 2013, 2014; Nowack and Bucheli 2007; Tolaymat et al. 2017). Metal and metal oxide nanoparticles are among the most widely used nanomaterials (Piccinno et al. 2012) and are known to negatively affect various aquatic organisms, including phytoplankton, the dominant primary producers in aquatic ecosystems (Likens 1975). These adverse outcomes are largely attributed to processes such as metal ion release, cellular adsorption and internalisation and the generation of reactive oxygen species (ROS) (Abdal Dayem et al. 2017; Aruoja et al. 2009). Although the magnitude of these effects is often species‐specific and sometimes nonsignificant (Kulacki and Cardinale 2012), negative outcomes at the species level have nevertheless been frequently documented (Aruoja et al. 2009; Bondarenko et al. 2013; Cortés‐Téllez et al. 2024; Das et al. 2014; Dedman et al. 2020; Miller et al. 2010). At the community level, studies have focused on how nanoparticles influence phytoplankton biomass and community structure (Conine et al. 2018; Dauda et al. 2020; Vincent et al. 2017; Wang et al. 2024), with limited attention to species interactions (Peng et al. 2017; Zuo et al. 2024). However, how biodiversity modulates the ecosystem‐level impacts of nanoparticles remains poorly understood.

Biodiversity enhances ecosystem functioning mainly through two mechanisms: complementarity, where species niche differentiation or facilitative interactions lead to greater functioning, and selection, where diverse communities are more likely to include species that strongly influence ecosystem processes (Loreau and Hector 2001; Tilman et al. 2014). A large body of research has identified complementarity as the dominant driver of net biodiversity effect (Cardinale et al. 2007; Chen et al. 2025; Fargione et al. 2007), particularly under climate stress (Hong et al. 2022). This pattern appears to align with the stress‐gradient hypothesis, which posits that facilitative species interactions become increasingly important as environmental stress intensifies (Bertness and Callaway 1994; Maestre et al. 2009). Nevertheless, the specific contribution of facilitative interactions to complementarity has rarely been explicitly disentangled in biodiversity‐ecosystem functioning studies. For the few studies on heavy metal pollution, biodiversity has been found to buffer productivity through either selection or complementarity effect (Awasthi et al. 2014; Li et al. 2010). Given that metal nanoparticles can also release toxic ions, it is reasonable to hypothesize that biodiversity may buffer their effects as well. However, nanoparticles can exert influences through other pathways, and once these are considered, it is likely that biodiversity effects may be undermined rather than enhanced. For example, phytoplankton species can complement each other in light utilisation through their partially non‐overlapping absorption spectra (Stomp et al. 2004). However, if the physical shading effect of nanoparticles plays a significant role in aquatic ecosystems (Gong et al. 2011; Wang et al. 2016), the spectrum of photosynthetically usable light may be narrowed, thereby reducing opportunities for spectral niche differentiation and weakening complementarity among phytoplankton species (Burson et al. 2019; Striebel et al. 2009). Additionally, more diverse communities are also more likely to include sensitive species that are prone to ROS‐induced oxidative stress (Abdal Dayem et al. 2017; Yu et al. 2020), potentially resulting in negative biodiversity effects on ecosystem functioning under nanoparticle exposure. These uncertainties underscore the need for empirical studies to clarify how biodiversity mediates the ecological impacts of nanoparticle exposure.

**FIGURE 1:**
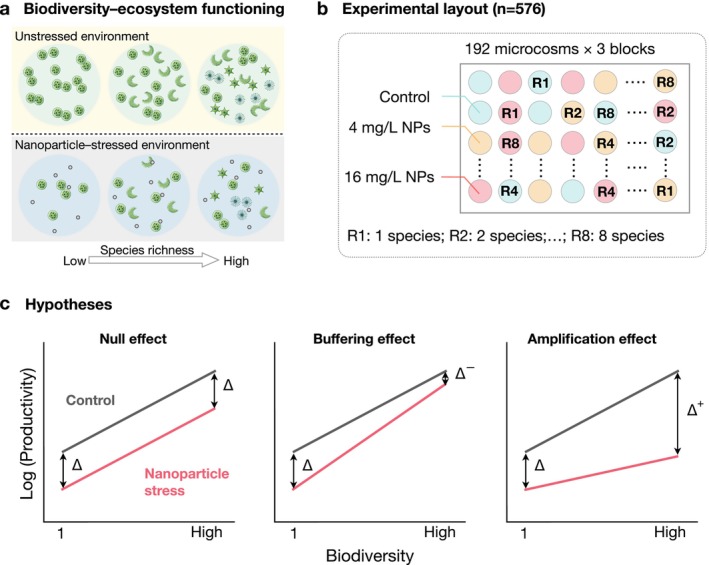
Diagrams illustrating (a) the central question addressed in this study, (b) the experimental design used to test our hypotheses, (c) the three hypothesized scenarios. Panel b shows the layout of experimental microcosms within a block. Three replicate blocks were established. Within each block, 192 microcosms were arranged across a gradient of species richness (1, 2, 4 and 8) and three nanoparticle concentrations (0, 4 and 16 mg/L). Panel c lists three possible scenarios of the biodiversity effects on ecosystem function under nanoparticle stress. The ‘Null effect’ is the null hypothesis that high‐diversity communities suffer similar influence from nanoparticles as low‐diversity communities. The other two alternative hypotheses are ‘buffering effect’ and ‘amplification effect’ of biodiversity in modulating the influence of nanoparticles on ecosystem function, respectively.

In this study, we experimentally subjected phytoplankton communities with varying species richness to a gradient of copper oxide nanoparticles to evaluate how biodiversity influences ecosystem‐level responses (Figure 1a,b). We measured biomass at both community and species levels to test three alternative hypotheses and identify the underlying mechanisms. Based on known variation in species sensitivity (Castro‐Bugallo et al. 2014; Miller et al. 2010; Zuo et al. 2024) and evidence that species interactions can shift under nanoparticle exposure (Peng et al. 2017; Zuo et al. 2024), we assumed that either complementarity or selection effects, or both, may be altered. The null effect hypothesis posits that changes in complementarity and selection effects do not scale systematically with species diversity, or tend to offset each other, resulting in a similar proportional decline in ecosystem functioning under nanoparticle exposure (Figure 1c). The buffering effect hypothesis predicts that high‐diversity communities are less negatively affected, driven by increased complementarity or the positive selection of tolerant, productive species (Figure 1c). In contrast, the amplification effect hypothesis suggests that more diverse communities experience disproportionately greater impairment, potentially driven by adverse shifts in complementarity or selection effects (Figure 1c).

## 2 Materials and Methods

### 2.1 Phytoplankton Species and Stock Cultures

We used eight green microalgal species obtained from Carolina Biology Supply (Burlington, NC, USA): *Ankistrodesmus falcatus* , *Chlamydomonas* sp., *Chlorella vulgaris* , *Haematococcus pluvialis* , *Nannochloris* sp., *Scenedesmus quadricauda* , *Selenastrum capricornutum* and *Staurastrum gracile* . We grew these species in the COMBO medium, which contains essential inorganic nutrients and vitamins that support algal growth (Kilham et al. 1998). The stock cultures of these species were unialgal but not axenic, while sterile medium, containers and sampling utensils were used for stock culture maintenance as well as for our experiments. We selected these species to form our species pool based on two criteria: first, their ability to grow and sustain viable populations in the COMBO medium; and second, their distinct morphological characteristics, which allow for species differentiation under a microscope and via flow cytometry. Prior to the experiment, all stock cultures were maintained inside an incubator set at 22°C, under a 12:12 light–dark cycle, with a light intensity of 50 μmol m^−2^ s^−1^. Biweekly, we transferred 100 μL of each stock culture to 100 mL fresh medium to maintain the exponential growth of the species.

### 2.2 CuO Nanoparticles

The CuO nanoparticles, whose nominal particle size is smaller than 50 nm, were purchased from Sigma‐Aldrich (St. Louis, MO, USA). We confirmed their characteristics and purity with scanning electron microscopy (SEM) and X‐ray diffraction (Figure S1a,b). After dispersion of CuO nanoparticles into the COMBO medium, we quantified their particle size distribution in the medium (Figure S1c) using a Zetasizer Nano (Malvern Panalytical, Malvern, UK).

### 2.3 Experimental Design and Sampling

We used a factorial design crossing four levels of species richness (1, 2, 4 and 8 species) with three nanoparticle treatments (control, 4 and 16 mg/L). All communities were assembled from the pool of the eight phytoplankton species. To account for species‐specific differences in productivity, all eight species were grown in monocultures (richness = 1; two replicates each). The composition of two‐ and four‐species communities was determined using a random partitioning design, yielding 16 unique communities at each richness level (Figure S2, Table S1). The eight‐species community, comprising the full species pool, was replicated 16 times (Figure S2, Table S1). The entire experiment was replicated across three independent blocks, each containing all communities and treatments, for a total of 576 microcosms (3 nanoparticle treatments × 4 richness levels × 16 communities × 3 blocks).

We prepared the COMBO medium following Kilham et al. (1998) and sterilised it by autoclaving at 121°C for 20 min. The medium was then rapidly cooled in a flowing cold‐water bath to minimise precipitation. The medium, which was prepared without additional buffering agents, had an initial pH of 7.87–7.92. We cultured each community in a 25 mL test tube containing 10 mL COMBO medium and capped it with an autoclavable polypropylene loose‐fitting push cap. All culture vessels and caps were autoclaved prior to use. At the start of the experiment, each microcosm was inoculated with 800 cells per mL, with an equal density allocated to each constituent species (i.e., 200 cells per species per mL in four‐species communities and 400 cells per species per mL in two‐species communities). The communities were cultured at 22°C in the incubators with a 12:12 light–dark cycle. To prevent nutrient depletion, we refreshed the medium weekly by removing 1 mL of culture and replacing it with 1 mL of fresh, autoclaved COMBO medium.

After initializing all the microcosms, we sampled the cultures weekly from Day 7 to 56. At each sampling point, cultures were thoroughly mixed using a sterile pipette to ensure homogeneity. A 200 μL sample was then extracted and transferred into a 96‐well microplate containing 10 μL of 1% sorbitol. The samples were kept in the dark at room temperature for 1 h to allow cell coating by sorbitol (Bestion et al. 2021) before being preserved at −80°C until flow cytometry analysis.

### 2.4 Data Acquisition and Analyses

#### 2.4.1 Community Composition and Biomass

We assayed the defrosted samples with a CytoFLEX S flow cytometer (Beckman Coulter, Brea, CA, USA), typically analysing 10 μL of culture per sample at a flow rate of 60 μL per min, and up to 20 μL for low‐density samples. All cells in the sample were detected, enumerated and recorded with a set of flow cytometric parameters (Table S2). Non‐cellular events were excluded by removing all flow records with PC7.H (far‐red fluorescence channel) values below 10,000 based on an empirical threshold indicating background noise or debris. Forward scatter height (FSC.H) was used as a proxy for cell size, based on an empirically derived linear relationship between FSC.H and species' equivalent spherical diameter (ESD; Figure S3; *R* ^2^ = 0.97, *p* < 0.001; see Text S1). A preliminary experiment confirmed that the presence of nanoparticles had no significant influence on FSC.H values (*p* = 0.88; Figure S4; see Text S1 for details). Therefore, we applied the FSC.H‐ESD relationship derived from the control monocultures to estimate cell size within communities across all nanoparticle treatments. Cell volume was subsequently converted to biomass using a published empirical volume‐to‐carbon relationship (Menden‐Deuer and Lessard 2000).

$$\text{biomass}\;\left(\mathrm{pg}\;C\;{\text{cell}}^{-1}\right)=0.216\times {\text{volume}}^{0.939}$$

For the samples of communities composed of at least two species, the species identity of phytoplankton cells was classified with a machine learning method. Briefly, for each nanoparticle treatment, we constructed a set of community‐specific classification models using the monoculture data of the component species and applied these models to classify individual cells within the corresponding communities (see more details in Text S2). The average overall accuracy is 99.1% for two‐species communities, 97.7% for four‐species communities and 96.5% for eight‐species communities (Table S3).

After calculating biomass and identifying species, we obtained weekly biomass data for each community from Day 7 to 56. By examining the temporal trajectories of monocultures, we identified Day 21 as a breakpoint when growth rates declined sharply and biomass reached quasi‐stationary levels (Figure S5). Therefore, we used the average biomass from Day 21 to 56 to represent the productivity of mature communities.

#### 2.4.2 Biodiversity Effects on Productivity

We quantified the net biodiversity effect (NE) and its two components, the complementarity effect (CE) and the selection effect (SE), using the additive partitioning method (Loreau and Hector 2001). Calculations were conducted separately for each block × nanoparticle treatment combination. Monoculture productivity of each species ($M_{i}$) was defined as the mean biomass from its two replicated monoculture microcosms. These species‐specific values ($M_{i}$) served as the references for computing biodiversity effects of each mixed‐species microcosm. For each species i in the mixture, the observed relative yield (${\mathrm{RY}}_{O,i}$) was calculated as the ratio of its biomass in mixture to its monoculture biomass ($M_{i}$). The expected relative yield (${\mathrm{RY}}_{E,i}$) was the species' initial proportion in the mixture (e.g., 1/8 for eight‐species communities, 1/4 for four‐species communities). $∆{\mathrm{RY}}_{i}$ was defined as ${\mathrm{RY}}_{O,i}-{\mathrm{RY}}_{E,i}$, and biodiversity effects were calculated as:

$$\mathrm{NE}=Y_{O}-Y_{E}=\sum_{i}{\mathrm{RY}}_{O,i}\;M_{i}-\sum_{i}{\mathrm{RY}}_{E,i}\;M_{i}=\sum_{i}∆{\mathrm{RY}}_{i}\;M_{i}=N\;\bar{∆\mathrm{RY}}\;\bar{M}+N\;\mathrm{cov}\left(∆{\mathrm{RY}}_{i},M_{i}\right)$$

$$\mathrm{CE}=N\;\bar{∆\mathrm{RY}}\;\bar{M}$$

$$\mathrm{SE}=N\;\mathrm{cov}\left(∆{\mathrm{RY}}_{i},M_{i}\right)$$

Here, $Y_{O}$ is the observed yield of the mixture, and $Y_{E}$ is the expected yield, calculated as the sum of species' expected yields under the null assumption of no complementary or selective effects. All biodiversity effects were expressed as relative values, calculated by dividing NE, CE and SE by the expected community yield ($Y_{E}$). This standardisation enables comparisons in biodiversity effect across treatments (Chen et al. 2025; Spake et al. 2023).

#### 2.4.3 Statistical Analyses

All statistical analyses were conducted in R 4.4.0 (R Core Team 2024). We analysed species biomass responses to nanoparticles using linear mixed‐effects (LME) models fitted with the lmer function in the lme4 package (Bates et al. 2015). The general model structure was:

$$Y_{\mathrm{ijk}}=\beta_{0}+\beta_{1}\sqrt{{\mathrm{NP}}_{i}}+b_{j}+c_{k}+\varepsilon_{\mathrm{ijk}}$$

where $Y_{\mathrm{ijk}}$ is the log‐transformed biomass of the focal species in the microcosm i, block j and community composition k, $\sqrt{{\mathrm{NP}}_{i}}$ is the square‐root‐transformed nanoparticle concentration, $b_{j}\sim N\left(0,\sigma_{b}^{2}\right)$ is the random intercept for block, $c_{k}\sim N\left(0,\sigma_{c}^{2}\right)$ is the random intercept for community composition (included only when a species occurred in multiple community compositions within a given richness level), and $\varepsilon_{\mathrm{ijk}}\sim N\left(0,\sigma^{2}\right)$ is the residual error. For monocultures and the eight‐species community, only the block random effect was included. Here, nanoparticle concentration was treated as a continuous predictor variable and was square‐root transformed to reduce the disproportionate influence of the highest concentration (Quinn and Keough 2002). We classified species with a significant negative response to nanoparticles as sensitive species and ranked their sensitivities by the absolute value of the estimated fixed‐effect coefficient for nanoparticle concentration. Species with non‐significant responses were considered as non‐sensitive species. We additionally fitted a generalised linear model (GLM) to assess the relationship between species' responses in monocultures and mixtures.

At the community level, we used LME models to assess how community biomass varied with species richness and nanoparticle treatments. In the first model, community biomass was the response variable, whereas log‐transformed species richness (continuous), nanoparticle treatment (categorical: control, 4 and 16 mg/L) and their interaction were treated as fixed effects and community composition was included as a random intercept. The model structure was:

$$Y_{\mathrm{ik}}=\beta_{0}+\beta_{1}\ln\left({\text{Richness}}_{i}\right)+\beta_{2}{\mathrm{NP}}_{i}+\beta_{3}\ln\left({\text{Richness}}_{i}\right)\times {\mathrm{NP}}_{i}+c_{k}+\varepsilon_{\mathrm{ik}}$$

where $Y_{\mathrm{ik}}$ is the log transformed community productivity of microcosm *i* within community *k*, $c_{k}\sim N\left(0,\sigma_{c}^{2}\right)$ is the random intercept for community composition and $\varepsilon_{\mathrm{ik}}\sim N\left(0,\sigma^{2}\right)$ is the residual error. After fitting this model, we used the emtrends function from the emmeans package (Lenth 2025) to estimate and compare the slopes of richness–community productivity relationships across nanoparticle treatments. To further compare community productivity among nanoparticle treatments within each richness level, we fitted a second model with a similar structure but specified both richness and nanoparticle treatment as categorical factors, including their interaction. Within each richness level, pairwise differences among nanoparticle treatments were evaluated using the contrast method in the emmeans package (Lenth 2025).

We analysed variation in biodiversity effects (NE, CE and SE) across nanoparticle treatments, both pooling all richness levels and within each richness level separately (2, 4 and 8). Models were specified as:

$$Y_{\mathrm{ik}}=\beta_{0}+\beta_{1}I{\left[\mathrm{NP}=4\right]}_{i}+\beta_{2}I{\left[\mathrm{NP}=16\right]}_{i}+c_{k}+\varepsilon_{\mathrm{ik}}$$

where $Y_{\mathrm{ik}}$ represents NE, CE, or SE of microcosm i within community composition k, $I{\left[\mathrm{NP}=4\right]}_{i}$ and $I{\left[\mathrm{NP}=16\right]}_{i}$ are dummy variables for nanoparticle treatments with control as the reference level, $c_{k}\sim N\left(0,\sigma_{c}^{2}\right)$ is the random intercept for community composition and $\varepsilon_{\mathrm{ik}}\sim N\left(0,\sigma^{2}\right)$ is the residual error. For richness levels without multiple community compositions, linear models without the random effect were fitted. We calculated a stress response intensity (SRI) for each community following Steudel et al. (2012):

$$\mathrm{SRI}=1-\frac{\bar{M_{i,\mathrm{nps}}}}{\bar{M_{i,0}}}$$

where $\bar{M_{i,0}}$ and $\bar{M_{i,\mathrm{nps}}}$ are the mean monoculture productivities of the component species of community i under control and nanoparticle treatments, respectively. We then examined the relationships between biodiversity effects (NE, CE and SE) and SRI using linear regressions.

To further explore the mechanisms behind CE and SE, we analysed nanoparticle effects on the observed relative yields of species within communities. Separate LME models were fitted for each richness level (2, 4 and 8). The general model structure was:

$$Y_{\mathrm{ijk}}=\beta_{0}+\beta_{1}{\mathrm{NP}}_{i}+s_{j}+c_{k}+\varepsilon_{\mathrm{ijk}}$$

where $Y_{\mathrm{ijk}}$ is the observed relative yield of species j in community k under nanoparticle treatment i, ${\mathrm{NP}}_{i}$ is the categorical fixed effect of nanoparticle treatment, $s_{j}\sim N\left(0,\sigma_{s}^{2}\right)$ is the random intercept for species identity, $c_{k}\sim N\left(0,\sigma_{c}^{2}\right)$ is the random intercept for community composition (included only when multiple compositions were present), and $\varepsilon_{\mathrm{ijk}}\sim N\left(0,\sigma^{2}\right)$ is the residual error. We classified a community as exhibiting facilitative species interactions if at least one species had an observed relative yield greater than one. The effect of nanoparticle exposure on the occurrence of facilitation was assessed using a chi‐square test.

Full model results, including parameter estimates, standard errors, confidence intervals and fit statistics, are provided in Table S4.

## 3 Results

### 3.1 Species Response to Nanoparticles

**FIGURE 2:**
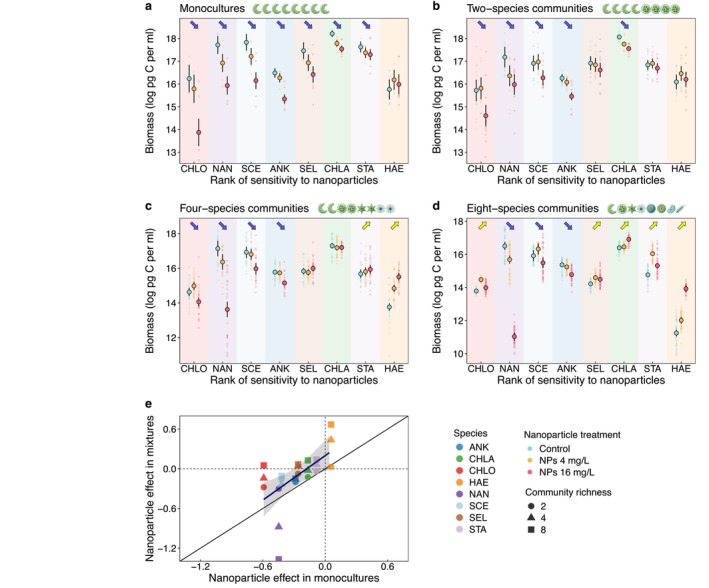
Species‐specific biomass responses to CuO nanoparticles when cultured as monocultures (a) or in communities with varying numbers of co‐occurring species (b–d), and the relationships between nanoparticle effects in mixtures and monocultures (e). In panels a–d, each small data point represents the biomass of a species in an individual microcosm, and large data points with error bars indicate the mean and standard error of species‐specific biomass for each nanoparticle treatment (control, 4 and 16 mg/L). Purple downward arrows and yellow upward arrows indicate statistically significant negative and positive effects of CuO nanoparticles, respectively. In panel e, the relationship was quantified using a generalised linear model, with the predicted relationship and 95% confidence interval shown. CHLO, *Chlorella vulgaris* ; NAN, *Nannochloris* sp.; SCE, *Scenedesmus quadricauda* ; ANK, *Ankistrodesmus falcatus* ; SEL, *Selenastrum capricornutum* ; CHLA, *Chlamydomonas* sp.; STA, *Staurastrum gracile* ; HAE, *Haematococcus pluvialis* .

Species‐specific responses to nanoparticle exposure depended on both the presence and the number of co‐occurring species. In monocultures, all species, except for *H. pluvialis* , exhibited negative responses, with the biomass of sensitive species declining by as much as 97% at the highest nanoparticle concentration (16 mg/L; Figure 2a). However, in multispecies communities, these negative effects were progressively alleviated as diversity increased, with some species in species‐rich assemblages even exhibiting positive responses (Figure 2b–d). While species responses in mixtures were positively correlated with their monoculture counterparts, most points fell above the 1:1 line (Figure 2e), again indicating that the negative effects of nanoparticles tended to be alleviated in mixtures.

### 3.2 Community Response to Nanoparticles

**FIGURE 3:**
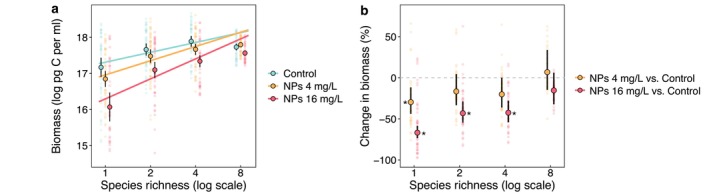
The biodiversity–productivity relationship under different nanoparticle concentrations (a), and nanoparticle‐induced changes in community productivity relative to the controls (b). In panel a, each small data point represents the biomass of a single microcosm, whereas large data points and error bars represent the mean and 95% confidence intervals of community biomass for each species richness and nanoparticle treatment combination. Solid lines indicate statistically significant positive relationships between biodiversity and productivity. In panel b, each small data point shows the percent difference in biomass between a nanoparticle‐treated community and its corresponding control community within the same block. Large data points and error bars indicate the mean and 95% confidence intervals of biomass changes for each species richness and nanoparticle treatment combination. Error bars that do not overlap with the horizontal zero line indicate statistically significant effects of nanoparticle pollution (*p* < 0.05), also denoted by asterisks.

Community biomass consistently increased with species richness across all nanoparticle treatments. However, the slope of this relationship became significantly steeper with nanoparticle addition (Figure 3a, Table S5). Compared to the controls, communities exposed to 4 and 16 mg/L nanoparticles exhibited reduced overall biomass, with the largest declines (averaging 30% and 67%, respectively) occurring at the lowest richness level. This negative effect weakened with increasing richness and became statistically non‐significant in eight‐species communities (Figure 3b).

### 3.3 Complementarity and Selection Effects, and Evidence for Facilitation

**FIGURE 4:**
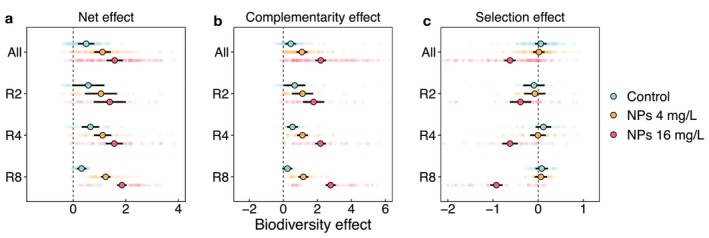
Net biodiversity effect (a) and its two components, complementarity effect (b) and selection effect (c). Small data points represent effects calculated for different microcosms. Large data points with error bars represent the means and 95% confidence intervals estimated for all communities (All), two‐species communities (R2), four‐species communities (R4) and eight‐species communities (R8). Error bars that do not overlap with the vertical zero line indicate significant positive or negative biodiversity effects.

Communities at all richness levels exhibited positive net biodiversity effects across all nanoparticle treatments, primarily driven by the positive complementarity effect (Figure 4a,b). The complementarity effect increased significantly with nanoparticle concentration, and this extent was amplified by species richness (Figure 4b, Table S6). In contrast, the selection effect, generally neutral under control and 4 mg/L nanoparticles, became significantly negative under 16 mg/L nanoparticle exposure (Figure 4c). Regression analyses showed that the net biodiversity effect and its complementarity component of multispecies (four and eight species) communities increased significantly with stress response intensity (SRI), although similar relationships were not observed for two‐species communities (Figure S6). By contrast, the selection effect decreased significantly with SRI across all communities (Figure S6).

**FIGURE 5:**
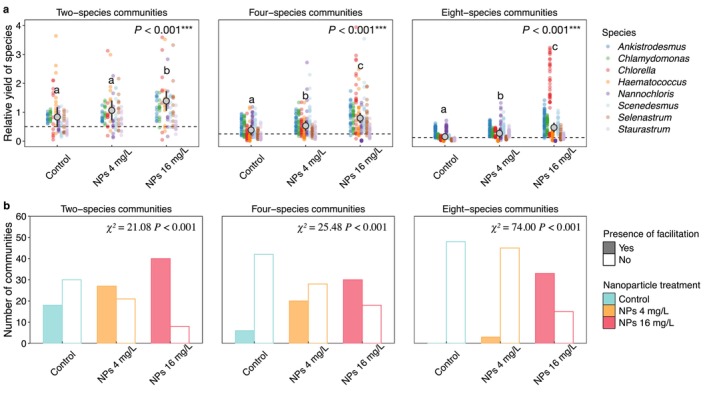
Species relative yield (a) and frequency of facilitation (b) across nanoparticle treatments under three different community richness levels. In panel a, dashed lines indicate the expected relative yield for each richness level (0.5 for two‐species, 0.25 for four‐species and 0.125 for eight‐species communities). Small coloured points represent individual species' relative yields within specific communities, and large grey points show means across all species and communities, with error bars indicating 95% confidence intervals. Different lowercase letters denote significant differences among nanoparticle treatments based on Tukey's HSD post hoc test. In panel b, bars show the number of communities that exhibited facilitation (filled) or did not (blank) under each nanoparticle treatment at each richness level. Results of chi‐squared tests (*χ* ^2^ and *p* values) are shown within the plots.

We further evaluated mechanisms underlying the complementarity effect by assessing relative yield and facilitation. Across communities of varying richness, relative yield increased consistently with nanoparticle concentration (Figure 5a), with species more negatively affected in monoculture tending to show higher relative yields in mixtures (Figure S7). The frequency of communities exhibiting facilitation also rose with nanoparticle concentration, and this effect was especially pronounced in species‐rich communities (Figure 5b).

## 4 Discussion

Nanoparticles pose well‐documented threats to organisms at both physiological and species levels (Aruoja et al. 2009; Dedman et al. 2020; Miller et al. 2010; Roy et al. 2014; Sukhanova et al. 2018), yet their impacts at community and ecosystem levels remain poorly understood. Our study provides unique experimental evidence that biodiversity can buffer phytoplankton communities against nanoparticle‐induced biomass loss, with diverse assemblages maintaining functioning through increased facilitation. These findings highlight the critical role of biodiversity in sustaining ecosystem functioning under novel chemical stressors.

### 4.1 Buffering Effect of Biodiversity Against Nanoparticle Pollution

The observed increase in biodiversity effects on biomass production with rising nanoparticle concentration (Figures 3a and 4a) provides direct support for the buffering effect hypothesis (Figure 1c), which suggests that biodiversity helps maintain ecosystem functioning under nanoparticle stress. Our results align with the increased biodiversity and ecosystem functioning (BEF) slopes under climatic stressors (Bestion et al. 2020, 2021) and traditional chemical pollutants (Awasthi et al. 2014; Li et al. 2010), and further extend the evidence to emerging chemical pollutants. This reinforces and broadens the generality of context dependency in BEF relationships, that is, biodiversity effects tend to be stronger and more critical under stressful conditions than in benign or favourable environments (Hong et al. 2022). The steeper positive slopes between biodiversity effects and stress intensity in species‐rich communities (Figure S6) provide additional evidence that the buffering effect of biodiversity becomes more pronounced with increasing species richness.

Beyond confirming our hypothesis, our findings offer further insight into how biodiversity loss may undermine ecosystem functioning. In particular, the steeper BEF relationships under nanoparticle exposure suggest less saturating biodiversity effects on biomass production (Reich et al. 2012). This pattern is particularly evident when standardised biomass (i.e., community biomass divided by the mean monoculture biomass) is plotted against species richness on arithmetic axes (Figure S8). Weaker saturation of the BEF relationship implies reduced functional redundancy, such that under nanoparticle stress the loss of even a few species in diverse communities may lead to substantial declines in ecosystem functioning (Reich et al. 2012; Rosenfeld 2002). Multiple environmental stressors have already accelerated biodiversity loss globally (Butchart et al. 2010; Lu et al. 2020; Pimm et al. 2014), particularly in regions with intense human activity (Blowes et al. 2019; Bowler et al. 2020). Without effective conservation strategies, continued biodiversity erosion together with increasing nanoparticle releases in these hotspots are likely to exacerbate functional declines in ecosystems.

### 4.2 Facilitation as a Driver of Increased Complementarity

The observed buffering effect of biodiversity can arise from either increased complementarity or selection effects (Loreau et al. 2001; Tilman et al. 2014). A selection‐driven buffering effect represents a statistical biodiversity effect, where functioning is maintained through the dominance of more productive and less sensitive species, while less productive and more sensitive species contribute little. In such cases, community biomass typically does not exceed that of the best‐performing monoculture. By contrast, a complementarity‐driven buffering effect reflects an interaction‐based biodiversity effect, where enhanced resource partitioning or facilitation among species can lead to transgressive overyielding—that is, a situation in which mixtures outperform even the most productive monoculture (Cardinale et al. 2007; Tilman et al. 2014). In our study, the buffering effect of biodiversity under nanoparticle stress was driven by a strong increase in complementarity, which more than compensated for the decline in the selection effect at high nanoparticle concentration (Figure 4).

In monocultures, *H. pluvialis* and *S. gracile* showed the least reduction in biomass among the eight species. The tolerance of *H. pluvialis* may stem from its production of astaxanthin, which scavenges reactive oxygen species (ROS) (Boussiba and Vonshak 1991; Zuluaga et al. 2018), and its multilayered cell wall that limits nanoparticle penetration (Hagen et al. 2002). *S. gracile'*s tolerance likely relates to its large cell volume and low surface area to volume ratio, which may reduce nanoparticle exposure (Zuo et al. 2024). In multispecies communities, both species showed increased biomass with increasing nanoparticle concentration (Figure 2c,d). For more sensitive species, the negative effects of nanoparticles were also buffered by neighbouring species. For example, *C. vulgaris* , the most sensitive species, showed substantial biomass reduction under nanoparticle exposure in monoculture, but this effect was progressively alleviated with increasing biodiversity, even reversing in eight‐species communities (Figure 2). Overall, although less sensitive species remained more resistant to nanoparticles in mixtures, biodiversity generally buffered the negative impacts across all species (Figure 2e), indicating a shift of species interaction towards less negative interactions. Furthermore, the overall increase in relative yield—particularly the more frequent occurrence of values exceeding one (i.e., species attaining greater biomass with than without co‐occurring species)—at elevated nanoparticle concentrations (Figure 5) provides strong evidence for enhanced facilitation. While previous studies have often reported increased complementarity under environmental stress (Hong et al. 2022), our study advances this understanding by identifying increased facilitation as a key driver of greater complementarity under nanoparticle stress. This finding is in line with the stress‐gradient hypothesis (Bertness and Callaway 1994; Maestre et al. 2009), and extends its applicability from frequently tested stressors to emerging chemical stressors such as nanoparticles.

Mechanistically, facilitative interactions observed under nanoparticle stress likely arose from two distinct but interlinked pathways. For relatively tolerant species, the suppression of sensitive species likely reduced nutrient competition, increasing resource availability and supporting growth under stress. This pathway is supported by the decline in the total biomass of the four most sensitive species in eight‐species communities under nanoparticle exposure (Figure S9). In addition, previous work has shown that nanoparticles can adhere to the surface of phytoplankton cells (Zhao et al. 2016), a finding corroborated by our SEM coupled with energy‐dispersive X‐ray spectroscopy (EDX) analysis, which revealed that CuO nanoparticles were visibly attached to cell surfaces (Figure S10). From the perspective of sensitive species, the presence of neighbouring taxa may have helped mitigate nanoparticle toxicity by adsorbing nanoparticles onto their surfaces, thereby reducing the concentration of free nanoparticles that would otherwise affect sensitive cells. Regression analysis further supports this pathway, showing that species more strongly suppressed by nanoparticles in monoculture tended to benefit more in diverse communities (Figure S7).

In contrast to the strengthening of complementarity by facilitation under nanoparticle stress, the selection effect showed the opposite pattern. A positive selection effect occurs when the best‐performing species contributes disproportionately to biomass production in mixtures, relative to its monoculture yield (Loreau and Hector 2001). However, such a pattern has not been consistently observed in previous BEF studies (Jiang et al. 2008), nor was it detected in our study. Instead, the selection effect shifted from neutral under control and 4 mg/L nanoparticle treatments to significantly negative under 16 mg/L nanoparticle exposure (Figure 4c). This negative selection effect appears to have been driven by the same facilitative interactions underlying the increased complementarity effect. In particular, under 16 mg/L nanoparticle exposure, species were re‐ranked in productivity based on their sensitivity, with more sensitive species performing poorly in monoculture but benefiting more from the presence of other species in mixtures. This shift led to a strong negative correlation between species' monoculture productivity and relative yield (Figure S11), which gave rise to a negative selection effect (Figure 4c). Nevertheless, although the selection effect turned negative under severe nanoparticle stress, the concurrent rise in complementarity resulted in an even stronger net biodiversity effect, reinforcing biodiversity's buffering role in sustaining ecosystem functioning under nanoparticle pollution.

### 4.3 Caveats

Two caveats should be considered when interpreting our findings. First, our experimental design did not explicitly account for the role of bacteria, which can act as decomposers and influence nutrient cycling, organic matter degradation and the release of dissolved organic compounds that in turn affect phytoplankton dynamics (Cole 1982; Ramanan et al. 2016). Such bacterial–phytoplankton interactions may have contributed to the observed biodiversity effects, either by modulating resource availability or by altering species interactions. Second, although we did not monitor pH dynamics throughout our experiment, a short‐term trial showed that medium pH increased as phytoplankton biomass accumulated, regardless of nanoparticle treatments (Figure S12). This pattern is consistent with phytoplankton uptake of dissolved CO_2_ during photosynthesis, which reduces carbonic acid and elevates medium pH (Wetzel 2001). Such pH shifts can alter nanoparticle dissolution, metal ion release and cellular uptake (Fernando and Zhou 2019; Franklin et al. 2000), potentially interacting with biodiversity effects. Future work that explicitly considers microbial communities and abiotic feedbacks such as pH will be important for a comprehensive understanding of how phytoplankton biodiversity mediates ecosystem functioning under nanoparticle stress.

## 5 Conclusion

Our study provides unique empirical evidence that biodiversity acts as an effective buffer for biomass production under nanoparticle pollution. Although nanoparticles negatively impacted most phytoplankton species in single‐species populations, increased biodiversity mitigated these impacts, preserving biomass in multispecies communities. This enhanced biodiversity effect was primarily driven by a strengthened complementarity effect, underpinned by facilitative species interactions that became more common under greater nanoparticle pollution. These findings highlight the important role of biodiversity in safeguarding ecosystem functioning against emerging chemical pollutants, providing a strong rationale for the conservation of biodiversity in the face of accelerating environmental change.

## Author Contributions

Yiping Zuo and Lin Jiang designed the project. Yiping Zuo carried out the experiment and analysed the data. Yiping Zuo and Lin Jiang wrote the manuscript. All authors contributed substantially to revisions and discussions.

## Supporting information


**Data S1:** ele70253‐sup‐0001‐Supinfo.pdf.

## Data Availability

The data and code supporting the findings of this study are openly available in Figshare at https://figshare.com/s/ceac3aed7508e385efdb.
