# Supplementary material for: Biodiversity Buffers Phytoplankton Biomass Production Against Nanoparticle Pollution Through Increased Facilitation
Source: Ecol Lett. 2025 Nov 9;28(11):e70253. doi: 10.1111/ele.70253 (PMC12597026; doi:10.1111/ele.70253)
Supplement: Supplementary file 1 — Data S1: ele70253‐sup‐0001‐Supinfo.pdf. [file ELE-28-0-s001.pdf]

Supplementary information for “Biodiversity buffers phytoplankton biomass production against nanoparticle pollution through increased facilitation”

Supplementary Text I. Biomass estimation from flow cytometric properties

To estimate phytoplankton biomass, we used forward scatter height (FSC.H) values obtained via flow cytometry as a proxy for cell size. FSC.H represents the peak intensity of forward scatter and is commonly used to approximate cell size in single-cell analysis (Koch *et al.* 1996).

We validated the reliability of FSC.H by assessing its relationship with equivalent spherical diameter (ESD) across species. For each species, we measured the dimensions of at least 20 cells under a compound microscope and calculated their volumes using morphology-specific geometric formulas (e.g., ellipsoid, cylinder) based on measured dimensions (Hillebrand *et al.* 1999). Because some species are not spherical, we converted volumes to ESDs to standardize size comparisons across morphologies. Regression analysis revealed a strong positive relationship between FSC.H and ESD ($R^2 = 0.97$, $P < 0.001$; Fig. S3), confirming the accuracy of FSC.H–based size estimation.

In the main experiment, this FSC.H–ESD relationship was used to estimate the ESD of each recorded cell by flow cytometry. These ESDs were then converted to cell volume and subsequently to biomass using the empirical volume–biomass relationship for non-diatom phytoplankton reported by Menden-Deuer & Lessard (2000):

$$B = 0.216 \times V^{0.939}$$

where B is biomass (pg C cell^{-1}) and V is cell volume (μm^3). The biomass of each individual cell was estimated in this way, and the summed biomass of all measured cells within a sample was divided by the analyzed sample volume to obtain community biomass.

Prior to applying this regression to samples exposed to nanoparticles, we conducted a preliminary experiment to assess whether the presence of copper oxide nanoparticles biases FSC.H–based size estimation. For each phytoplankton species, we prepared six identical monoculture samples (100 μL each). Three were mixed with 100 μL of pure COMBO medium (control), and the other three with 100 μL of COMBO containing 32 mg/L CuO nanoparticles, yielding final nanoparticle concentrations of 0 and 16 mg/L, respectively. Samples were briefly vortexed and incubated in the dark for one hour before FSC.H measurement via flow cytometry. A linear mixed-effects model (FSC.H ~ nanoparticle treatment + (1 | species)) was used to test for treatment effects. The model revealed no significant difference between treatments ($P = 0.88$; Fig. S4), indicating that nanoparticles did not interfere with FSC.H–based size estimation under these short-term exposure conditions. We note that this test was not designed to evaluate long-term effects of nanoparticles on cell size, but rather to confirm that their presence does not bias flow cytometric size estimation during analysis.

Supplementary Text II. Species discrimination in communities

To determine species identity of individual cells in communities composed of two or more species, we employed a supervised machine learning approach using random forest classification trained on monoculture data. Specifically, for each unique community composition under each nanoparticle treatment, we constructed a separate classification model tailored to the corresponding species combination.

Species identity was assigned to each cell in the monoculture data and used as the response variable. A total of 18 flow cytometric features were used as predictors: FSC.H, FSC.A, FSC.Width, SSC.H, SSC.A, FITC.H, PerCP.H, APC.H, APC_A700.H, APC_A750.H, PB450.H, KO525.H, Violet610.H, Violet660.H, PE.H, ECD.H, PC5.5.H, and PC7.H. All the features were natural log-transformed prior to model training to reduce skewness and improve classification performance.

For each model, we split the monoculture data of the focal species into a 75% training set and a 25% testing set. The training set was used to train a random forest classifier using the randomForest package (Liaw & Wiener 2002) in R (R Core Team 2024), with 500 trees. The testing set was used to evaluate classification accuracy, with results reported in Table S3.

References

Hillebrand, H., Dürselen, C.-D., Kirschtel, D., Pollingher, U. & Zohary, T. (1999). Biovolume calculation for pelagic and benthic microalgae. *J. Phycol.*, 35, 403–424.

Koch, A.L., Robertson, B.R. & Button, D.K. (1996). Deduction of the cell volume and mass from forward scatter intensity of bacteria analyzed by flow cytometry. *J. Microbiol. Methods*, 27, 49–61.

Liaw, A. & Wiener, M. (2002). Classification and regression by randomForest. R News, 2, 18–22.

Menden-Deuer, S. & Lessard, E.J. (2000). Carbon to volume relationships for dinoflagellates, diatoms, and other protist plankton. *Limnol. Oceanogr.*, 45, 569–579.

R Core Team (2024). R: A language and environment for statistical computing. R Foundation for Statistical Computing, Vienna, Austria.

Supplementary tables

Table S1 Species composition of phytoplankton assemblages used in the present study. For each of the nanoparticle treatments and blocks, the identical set of assemblages was used. The species name of corresponding ID: Species 1: *Ankistrodesmus falcatus*, Species 2: *Chlamydomonas sp.*, Species 3: *Chlorella vulgaris*, Species 4: *Haematococcus pluvialis*, Species 5: *Nannochloris sp.*, Species 6: *Scenedesmus quadricauda*, Species 7: *Selenastrum capricornutum*, Species 8: *Staurastrum gracile*.

Assemblage	community	richness	Assemblage replication	Total
1	sp_1	1	2	8 assemblages × 2 replicates = 16 microcosms
2	sp_2	1	2	
3	sp_3	1	2	
4	sp_4	1	2	
5	sp_5	1	2	
6	sp_6	1	2	
7	sp_7	1	2	
8	sp_8	1	2	
9	sp_1_8	2	1	16 assemblages × 1 replicate = 16 microcosms
10	sp_2_7	2	1	
11	sp_5_6	2	1	
12	sp_3_4	2	1	
13	sp_3_6	2	1	
14	sp_2_8	2	1	
15	sp_1_4	2	1	
16	sp_5_7	2	1	
17	sp_4_8	2	1	
18	sp_1_2	2	1	
19	sp_6_7	2	1	
20	sp_3_5	2	1	
21	sp_1_6	2	1	
22	sp_5_8	2	1	
23	sp_3_7	2	1	
24	sp_2_4	2	1	
25	sp_1_6_7_8	4	1	16 assemblages × 1 replicate = 16 microcosms
26	sp_2_3_4_5	4	1	
27	sp_3_4_6_7	4	1	
28	sp_1_2_5_8	4	1	
29	sp_4_6_7_8	4	1	
30	sp_1_2_3_5	4	1	
31	sp_2_4_5_6	4	1	
32	sp_1_3_7_8	4	1	
33	sp_1_2_6_8	4	1	
34	sp_3_4_5_7	4	1	
35	sp_1_4_5_6	4	1	

36	sp_2_3_7_8	4	1	
37	sp_3_4_5_6	4	1	
38	sp_1_2_7_8	4	1	
39	sp_1_3_5_7	4	1	
40	sp_2_4_6_8	4	1	
41	sp_1_2_3_4_5_6_7_8	8	16	1 assemblage × 16 replicates = 16 microcosms

Table S2 Summary of the scatter and fluorescence channels used in this study. Original variable refers to internal flow cytometry variable names; actual channel label corresponds to the fluorophore or scatter signal measured on the CytoFLEX platform. Descriptions are common fluorophores or emission ranges associated with each channel.

Original Variable	Actual channel label	Description
FSC	FSC	Forward Scatter (cell size proxy)
SSC	SSC	Side Scatter (cell granularity/complexity)
FL1	FITC	Fluorescein isothiocyanate (green, 525/40 nm)
FL2	PerCP	Peridinin-Chlorophyll-Protein (676/29 nm)
FL3	APC	Allophycocyanin (660/20 nm)
FL4	APC_A700	APC-Alexa Fluor 700 tandem (712/25 nm)
FL5	APC_A750	APC-Alexa Fluor 750 tandem (780/60 nm)
FL6	PB450	Pacific Blue (450/45 nm)
FL7	K0525	Krome Orange (525/40 nm)
FL8	Violet610	Violet-excited dye (610/20 nm)
FL9	Violet660	Violet-excited dye (660/20 nm)
FL10	PE	Phycoerythrin (575/26 nm)
FL11	ECD	PE-Texas Red (610/20 nm)
FL12	PC5.5	PE-Cyanine 5.5 (695/30 nm)
FL13	PC7	PE-Cyanine 7 (780/60 nm)

Table S3 Accuracies of random forest models used to discriminate species composition in (a) two-species communities, (b) four-species communities, and (c) eight-species communities. Each row represents a unique community, with species names listed in order and separated by underscores. The species names in the community label correspond in order to the prediction accuracy columns (e.g., the first species listed corresponds to “species 1”, the second to “species 2”). Accuracy values indicate model performance in predicting the identity of each species. The "Mean" column represents the average prediction accuracy across all species in the community. Species abbreviations: ANK, *Ankistrodesmus falcatus*, CHLA, *Chlamydomonas sp.*, CHLO, *Chlorella vulgaris*, HAE, *Haematococcus pluvialis*, NAN, *Nannochloris sp.*, SCE, *Scenedesmus quadricauda*, SEL, *Selenastrum capricornutum*, STA, *Staurastrum gracile*.

(a)

Community	Control			NPs 4 mg/L			NPs 16 mg/L		
	species 1	species 2	Mean	species 1	species 2	Mean	species 1	species 2	Mean
ANK_CHLA	0.998	0.993	0.996	0.999	0.991	0.995	0.992	0.995	0.993
ANK_HAE	0.999	0.998	0.999	1.000	0.996	0.998	0.996	0.998	0.997
ANK_SCE	1.000	0.998	0.999	1.000	0.997	0.998	0.997	0.996	0.997
ANK_STA	1.000	0.984	0.992	1.000	0.980	0.990	0.994	0.988	0.991
CHLA_HAE	0.996	0.992	0.994	0.996	0.985	0.991	0.993	0.991	0.992
CHLA_SEL	0.997	0.999	0.998	0.996	0.992	0.994	0.997	0.996	0.996
CHLA_STA	0.994	0.994	0.994	0.993	0.997	0.995	0.991	0.991	0.991
CHLO_HAE	0.985	0.987	0.986	0.979	0.979	0.979	0.966	0.973	0.969
CHLO_NAN	0.964	0.982	0.973	0.937	0.981	0.959	0.929	0.962	0.945
CHLO_SCE	0.998	0.999	0.998	0.998	0.996	0.997	0.999	0.998	0.998
CHLO_SEL	0.992	0.991	0.991	0.991	0.975	0.983	0.979	0.988	0.984
HAE_STA	0.997	0.987	0.992	0.998	0.991	0.995	0.996	0.991	0.994
NAN_SCE	0.998	0.997	0.997	1.000	0.996	0.998	0.999	0.996	0.998
NAN_SEL	0.992	0.983	0.987	0.993	0.974	0.984	0.995	0.991	0.993
NAN_STA	0.996	0.982	0.989	0.998	0.984	0.991	0.996	0.995	0.996
SCE_SEL	0.998	0.999	0.999	0.999	0.999	0.999	0.996	1.000	0.998

(b)

Community	Control					NPs 4 mg/L					NPs 16 mg/L				
	species 1	species 2	species 3	species 4	Mean	species 1	species 2	species 3	species 4	Mean	species 1	species 2	species 3	species 4	Mean
ANK_CHLA_CHLO_NAN	0.997	0.982	0.948	0.974	0.975	0.998	0.982	0.925	0.969	0.968	0.985	0.985	0.875	0.941	0.947
ANK_CHLA_NAN_STA	0.998	0.985	0.984	0.982	0.987	0.998	0.978	0.986	0.981	0.986	0.988	0.983	0.978	0.979	0.982

ANK_CHLA_SCE_STA	0.998	0.987	0.996	0.983	0.991	0.999	0.985	0.990	0.988	0.990	0.992	0.990	0.989	0.976	0.987
ANK_CHLA_SEL_STA	0.997	0.988	0.995	0.990	0.992	0.998	0.984	0.982	0.986	0.987	0.984	0.990	0.993	0.969	0.984
ANK_CHLO_NAN_SEL	0.997	0.955	0.979	0.975	0.977	0.998	0.940	0.977	0.944	0.965	0.990	0.893	0.967	0.981	0.958
ANK_CHLO_SEL_STA	0.997	0.988	0.988	0.976	0.987	0.999	0.979	0.965	0.980	0.981	0.986	0.947	0.987	0.985	0.976
ANK_HAE_NAN_SCE	0.998	0.972	0.972	0.995	0.984	0.999	0.968	0.976	0.992	0.983	0.993	0.959	0.969	0.995	0.979
ANK_SCE_SEL_STA	0.997	0.995	0.995	0.976	0.991	0.999	0.992	0.987	0.988	0.991	0.982	0.993	0.995	0.981	0.988
CHLA_CHLO_HAE_NAN	0.985	0.951	0.960	0.958	0.963	0.987	0.925	0.950	0.959	0.955	0.987	0.901	0.939	0.949	0.944
CHLA_CHLO_SEL_STA	0.990	0.981	0.987	0.988	0.987	0.990	0.967	0.963	0.981	0.975	0.986	0.947	0.985	0.985	0.976
CHLA_HAE_NAN_SCE	0.990	0.968	0.969	0.994	0.980	0.988	0.960	0.969	0.995	0.978	0.991	0.953	0.954	0.995	0.973
CHLA_HAE_SCE_STA	0.992	0.990	0.994	0.987	0.991	0.989	0.985	0.993	0.974	0.985	0.988	0.988	0.994	0.988	0.989
CHLO_HAE_NAN_SCE	0.955	0.964	0.965	0.993	0.969	0.928	0.957	0.965	0.994	0.961	0.918	0.945	0.947	0.992	0.950
CHLO_HAE_NAN_SEL	0.957	0.965	0.969	0.972	0.966	0.937	0.957	0.967	0.938	0.950	0.926	0.943	0.957	0.978	0.951
CHLO_HAE_SCE_SEL	0.983	0.983	0.995	0.987	0.987	0.974	0.974	0.992	0.964	0.976	0.950	0.970	0.992	0.985	0.974
HAE_SCE_SEL_STA	0.993	0.995	0.993	0.982	0.990	0.992	0.992	0.985	0.988	0.989	0.990	0.994	0.988	0.987	0.989

(c)

Community	Nanoparticle treatment	species 1	species 2	species 3	species 4	species 5	species 6	species 7	species 8	Mean
ANK_CHLA_CHLO_HAE_NAN_SCE_SEL_STA	Control	0.996	0.976	0.948	0.964	0.961	0.992	0.967	0.981	0.973
	NPs 4 mg/L	0.997	0.976	0.923	0.947	0.962	0.990	0.929	0.952	0.959
	NPs 16 mg/L	0.984	0.983	0.902	0.931	0.953	0.989	0.973	0.977	0.961

Table S4 Summary of the main linear mixed-effects (LME) and generalized linear (GLM) models fitted in this study. Fixed effects that are statistically significant are shown in bold. Richness was log-transformed when treated as a continuous predictor, and nanoparticle treatment was square-root transformed when treated as a continuous predictor. Species and community biomass were log-transformed as response variables. When modeled as categorical predictors, Richness = 1 species and the nanoparticle control treatment (0 mg/L) served as the reference. For LME models, R^2m and R^2c represent marginal and conditional R^2, respectively.

Model number	Response variable	Richness level	Fixed effect					Random effects	R2
			Term	Estimate	SE	95% CI		Variance ± SD	
**Models of species-specific biomass in monocultures**									
1	ANK biomass	R=1	Intercept<br>**sqrt(NPs)**	16.61<br>**-0.29**	0.20<br>**0.04**	16.21<br>**-0.37**	17.01<br>**-0.20**	block:0.09 ± 0.30	R^2m 0.57<br>R^2c 0.79
2	CHLA biomass	R=1	Intercept<br>**sqrt(NPs)**	18.18<br>**-0.17**	0.14<br>**0.02**	17.90<br>**-0.21**	18.46<br>**-0.13**	block:0.05 ± 0.23	R^2m 0.52<br>R^2c 0.87
3	CHLO biomass	R=1	Intercept<br>**sqrt(NPs)**	16.45<br>**-0.59**	0.62<br>**0.11**	15.24<br>**-0.81**	17.67<br>**-0.37**	block:0.90 ± 0.95	R^2m 0.41<br>R^2c 0.77
4	HAE biomass	R=1	Intercept<br>sqrt(NPs)	15.86<br>0.06	0.44<br>0.05	15.01<br>-0.05	16.72<br>0.16	block:0.52 ± 0.72	R^2m 0.01<br>R^2c 0.80
5	NAN biomass	R=1	Intercept<br>**sqrt(NPs)**	17.75<br>**-0.45**	0.39<br>**0.05**	16.99<br>**-0.55**	18.52<br>**-0.34**	block:0.40 ± 0.63	R^2m 0.51<br>R^2c 0.88
6	SCE biomass	R=1	Intercept<br>**sqrt(NPs)**	17.90<br>**-0.42**	0.36<br>**0.05**	17.19<br>**-0.53**	18.62<br>**-0.31**	block:0.34 ± 0.58	R^2m 0.51<br>R^2c 0.85
7	SEL biomass	R=1	Intercept<br>**sqrt(NPs)**	17.46<br>**-0.26**	0.36<br>**0.06**	16.76<br>**-0.38**	18.16<br>**-0.14**	block:0.31 ± 0.56	R^2m 0.28<br>R^2c 0.74
8	STA biomass	R=1	Intercept<br>**sqrt(NPs)**	17.60<br>**-0.08**	0.23<br>**0.04**	17.15<br>**-0.17**	18.05<br>**0.00**	block:0.12 ± 0.35	R^2m 0.09<br>R^2c 0.61
**Models of species-specific biomass in two-species communities**									
9	ANK biomass	R=2	Intercept<br>**sqrt(NPs)**	16.33<br>**-0.20**	0.17<br>**0.03**	15.99<br>**-0.25**	16.67<br>**-0.15**	community:0.02 ± 0.15<br>block:0.06 ± 0.24	R^2m 0.43<br>R^2c 0.74
10	CHLA biomass	R=2	Intercept<br>**sqrt(NPs)**	18.05<br>**-0.13**	0.07<br>**0.02**	17.91<br>**-0.16**	18.19<br>**-0.09**	community:0.01 ± 0.08<br>block:0.00 ± 0.06	R^2m 0.51<br>R^2c 0.62
11	CHLO biomass	R=2	Intercept<br>**sqrt(NPs)**	15.94<br>**-0.28**	0.46<br>**0.10**	15.03<br>**-0.47**	16.85<br>**-0.09**	community:0.50 ± 0.71<br>block:0.08 ± 0.28	R^2m 0.12<br>R^2c 0.46
12	HAE biomass	R=2	Intercept<br>sqrt(NPs)	16.20<br>0.03	0.32<br>0.05	15.57<br>-0.06	16.82<br>0.12	community:0.25 ± 0.50<br>block:0.07 ± 0.27	R^2m 0.00<br>R^2c 0.61
13	NAN biomass	R=2	Intercept<br>**sqrt(NPs)**	17.11<br>**-0.30**	0.45<br>**0.05**	16.23<br>**-0.39**	17.99<br>**-0.21**	community:0.31 ± 0.55<br>block:0.34 ± 0.58	R^2m 0.23<br>R^2c 0.82
14	SCE biomass	R=2	Intercept	17.03	0.34	16.37	17.70	community:0.17 ± 0.41	R^2m 0.12

			**sqrt(NPs)**	**-0.16**	**0.04**	**-0.24**	**-0.08**	block:0.19 ± 0.43	R^2c 0.72
15	SEL biomass	R=2	Intercept<br>sqrt(NPs)	16.94<br>-0.07	0.30<br>0.04	16.35<br>-0.16	17.54<br>0.01	community:0.13 ± 0.36<br>block:0.14 ± 0.37	R^2m 0.03<br>R^2c 0.60
16	STA biomass	R=2	Intercept<br>sqrt(NPs)	16.89<br>-0.04	0.21<br>0.03	16.47<br>-0.10	17.30<br>0.03	community:0.11 ± 0.33<br>block:0.03 ± 0.17	R^2m 0.01<br>R^2c 0.56
**Models of species-specific biomass in four-species communities**									
17	ANK biomass	R=4	Intercept<br>**sqrt(NPs)**	15.87<br>**-0.16**	0.13<br>**0.02**	15.60<br>**-0.20**	16.13<br>**-0.11**	community:0.12 ± 0.35<br>block:0.00 ± 0.00	R^2m 0.24<br>R^2c 0.66
18	CHLA biomass	R=4	Intercept<br>sqrt(NPs)	17.28<br>-0.02	0.16<br>0.02	16.97<br>-0.07	17.59<br>0.02	community:0.17 ± 0.41<br>block:0.00 ± 0.06	R^2m 0.01<br>R^2c 0.67
19	CHLO biomass	R=4	Intercept<br>**sqrt(NPs)**	14.84<br>**-0.14**	0.24<br>**0.05**	14.37<br>**-0.24**	15.31<br>**-0.04**	community:0.06 ± 0.24<br>block:0.10 ± 0.32	R^2m 0.08<br>R^2c 0.31
20	HAE biomass	R=4	Intercept<br>**sqrt(NPs)**	13.83<br>**0.44**	0.21<br>**0.04**	13.41<br>**0.35**	14.25<br>**0.52**	community:0.26 ± 0.51<br>block:0.00 ± 0.00	R^2m 0.44<br>R^2c 0.67
21	NAN biomass	R=4	Intercept<br>**sqrt(NPs)**	17.47<br>**-0.88**	0.44<br>**0.09**	16.60<br>**-1.05**	18.33<br>**-0.71**	community:0.26 ± 0.50<br>block:0.34 ± 0.58	R^2m 0.50<br>R^2c 0.65
22	SCE biomass	R=4	Intercept<br>**sqrt(NPs)**	17.04<br>**-0.24**	0.32<br>**0.03**	16.41<br>**-0.30**	17.67<br>**-0.18**	community:0.31 ± 0.56<br>block:0.18 ± 0.42	R^2m 0.19<br>R^2c 0.79
23	SEL biomass	R=4	Intercept<br>sqrt(NPs)	15.78<br>0.04	0.17<br>0.03	15.44<br>-0.02	16.12<br>0.10	community:0.06 ± 0.25<br>block:0.05 ± 0.22	R^2m 0.01<br>R^2c 0.40
24	STA biomass	R=4	Intercept<br>**sqrt(NPs)**	15.68<br>**0.06**	0.24<br>**0.03**	15.20<br>**0.01**	16.15<br>**0.12**	community:0.38 ± 0.62<br>block:0.02 ± 0.13	R^2m 0.02<br>R^2c 0.75
**Models of species-specific biomass in eight-species communities**									
25	ANK biomass	R=8	Intercept<br>**sqrt(NPs)**	15.43<br>**-0.15**	0.12<br>**0.02**	15.19<br>**-0.18**	15.67<br>**-0.11**	block:0.04 ± 0.20	R^2m 0.28<br>R^2c 0.47
26	CHLA biomass	R=8	Intercept<br>**sqrt(NPs)**	16.33<br>**0.13**	0.06<br>**0.01**	16.22<br>**0.11**	16.45<br>**0.15**	block:0.01 ± 0.09	R^2m 0.44<br>R^2c 0.53
27	CHLO biomass	R=8	Intercept<br>**sqrt(NPs)**	13.98<br>**0.05**	0.07<br>**0.02**	13.85<br>**0.02**	14.12<br>**0.08**	block:0.01 ± 0.09	R^2m 0.06<br>R^2c 0.12
28	HAE biomass	R=8	Intercept<br>**sqrt(NPs)**	11.06<br>**0.67**	0.20<br>**0.03**	10.67<br>**0.62**	11.45<br>**0.72**	block:0.11 ± 0.32	R^2m 0.77<br>R^2c 0.84
29	NAN biomass	R=8	Intercept<br>**sqrt(NPs)**	17.15<br>**-1.37**	0.26<br>**0.06**	16.64<br>**-1.48**	17.65<br>**-1.25**	block:0.13 ± 0.36	R^2m 0.78<br>R^2c 0.80
30	SCE biomass	R=8	Intercept<br>**sqrt(NPs)**	16.13<br>**-0.11**	0.33<br>**0.02**	15.48<br>**-0.15**	16.77<br>**-0.06**	block:0.31 ± 0.56	R^2m 0.05<br>R^2c 0.60
31	SEL biomass	R=8	Intercept<br>**sqrt(NPs)**	14.30<br>**0.07**	0.08<br>**0.02**	14.14<br>**0.03**	14.47<br>**0.10**	block:0.02 ± 0.12	R^2m 0.09<br>R^2c 0.19
32	STA biomass	R=8	Intercept	15.10	0.12	14.86	15.34	block:0.02 ± 0.15	R^2m 0.10

			**sqrt(NPs)**	**0.14**	**0.03**	**0.07**	**0.21**		R^2c 0.15
**Models of community biomass (Richness as continuous variable, nanoparticle treatment as categorical variable)**									
33	Community biomass	All	(Intercept)	17.31	0.17	16.97	17.65	community:0.31 ± 0.56	R^2m 0.35
			**NPs = 4 mg/L**	**-0.35**	**0.08**	**-0.51**	**-0.20**		R^2c 0.73
			**NPs = 16 mg/L**	**-1.02**	**0.08**	**-1.17**	**-0.86**		
			**log(richness)**	**0.39**	**0.17**	**0.07**	**0.72**		
			**NPs (4 mg/L) × log(Richness)**	**0.17**	**0.06**	**0.05**	**0.30**		
			**NPs (16 mg/L) × log(Richness)**	**0.41**	**0.06**	**0.28**	**0.53**		
**Models of community biomass (Both richness and nanoparticle treatment as categorical variables)**									
34	Community biomass	All	(Intercept)	17.17	0.21	16.76	17.58	community:0.31 ± 0.56	R^2m 0.31
			**NPs = 4 mg/L**	**-0.35**	**0.10**	**-0.54**	**-0.16**		R^2c 0.72
			**NPs = 16 mg/L**	**-1.10**	**0.10**	**-1.29**	**-0.91**		
			Richness = 2	0.49	0.26	-0.02	1.00		
			**Richness = 4**	**0.72**	**0.26**	**0.21**	**1.23**		
			Richness = 8	0.56	0.60	-0.61	1.74		
			NPs (4 mg/L) × Richness (2)	0.17	0.14	-0.10	0.44		
			**NPs (16 mg/L) × Richness (2)**	**0.54**	**0.14**	**0.27**	**0.80**		
			NPs (4 mg/L) × Richness (4)	0.13	0.14	-0.14	0.39		
			**NPs (16 mg/L) × Richness (4)**	**0.55**	**0.14**	**0.28**	**0.81**		
			**NPs (4 mg/L) × Richness (8)**	**0.42**	**0.14**	**0.15**	**0.68**		
			**NPs (16 mg/L) × Richness (8)**	**0.93**	**0.14**	**0.67**	**1.20**		
**Models of biodiversity effects (net biodiversity effects, complementarity effect, and selection effect)**									
35	Net biodiversity effect	R=2	(Intercept)	0.57	0.29	0.00	1.15	community:1.07 ± 1.04	R^2m 0.05
			**NPs = 4 mg/L**	**0.48**	**0.20**	**0.10**	**0.87**		R^2c 0.56
			**NPs = 16 mg/L**	**0.81**	**0.20**	**0.43**	**1.20**		
36	Net biodiversity effect	R=4	(Intercept)	0.65	0.16	0.34	0.97	community:0.27 ± 0.52	R^2m 0.17
			**NPs = 4 mg/L**	**0.46**	**0.13**	**0.20**	**0.73**		R^2c 0.48
			**NPs = 16 mg/L**	**0.91**	**0.13**	**0.64**	**1.17**		
37	Net biodiversity effect	R=8	(Intercept)	0.32	0.09	0.14	0.50		0.50
			**NPs = 4 mg/L**	**0.92**	**0.13**	**0.66**	**1.17**		
			**NPs = 16 mg/L**	**1.53**	**0.13**	**1.28**	**1.79**		
38	Net biodiversity effect	All	(Intercept)	0.49	0.15	0.19	0.80	community:0.63 ± 0.80	R^2m 0.14
			**NPs = 4 mg/L**	**0.62**	**0.09**	**0.44**	**0.80**		R^2c 0.58
			**NPs = 16 mg/L**	**1.09**	**0.09**	**0.91**	**1.26**		
39	Complementarity effect	R=2	(Intercept)	0.67	0.30	0.07	1.27	community:1.01 ± 1.00	R^2m 0.08
			NPs = 4 mg/L	0.46	0.24	-0.02	0.94		R^2c 0.46
			**NPs = 16 mg/L**	**1.11**	**0.24**	**0.63**	**1.59**		
40		R=4	(Intercept)	0.54	0.16	0.23	0.85	community:0.11 ± 0.34	R^2m 0.33

	Complementarity effect		**NPs = 4 mg/L**<br>**NPs = 16 mg/L**	**0.59**<br>**1.65**	**0.19**<br>**0.19**	**0.22**<br>**1.28**	**0.95**<br>**2.01**		R^2c 0.41
41	Complementarity effect	R=8	(Intercept)<br>**NPs = 4 mg/L**<br>**NPs = 16 mg/L**	0.24<br>**0.94**<br>**2.54**	0.15<br>**0.21**<br>**0.21**	-0.05<br>**0.52**<br>**2.12**	0.53<br>**1.35**<br>**2.95**	NA	0.52
42	Complementarity effect	All	(Intercept)<br>**NPs = 4 mg/L**<br>**NPs = 16 mg/L**	0.44<br>**0.66**<br>**1.77**	0.16<br>**0.13**<br>**0.13**	0.13<br>**0.41**<br>**1.52**	0.74<br>**0.91**<br>**2.01**	community:0.51 ± 0.72	R^2m 0.24<br>R^2c 0.47
43	Selection effect	R=2	(Intercept)<br>NPs = 4 mg/L<br>**NPs = 16 mg/L**	-0.09<br>0.02<br>**-0.30**	0.12<br>0.13<br>**0.13**	-0.32<br>-0.23<br>**-0.55**	0.13<br>0.27<br>**-0.05**	community:0.09 ± 0.29	R^2m 0.04<br>R^2c 0.21
44	Selection effect	R=4	(Intercept)<br>NPs = 4 mg/L<br>**NPs = 16 mg/L**	0.12<br>-0.12<br>**-0.74**	0.09<br>0.10<br>**0.10**	-0.05<br>-0.32<br>**-0.94**	0.28<br>0.08<br>**-0.54**	community:0.03 ± 0.18	R^2m 0.27<br>R^2c 0.35
45	Selection effect	R=8	(Intercept)<br>NPs = 4 mg/L<br>**NPs = 16 mg/L**	0.08<br>-0.02<br>**-1.00**	0.07<br>0.10<br>**0.10**	-0.06<br>-0.21<br>**-1.20**	0.21<br>0.17<br>**-0.81**	NA	0.50
46	Selection effect	All	(Intercept)<br>NPs = 4 mg/L<br>**NPs = 16 mg/L**	0.06<br>-0.04<br>**-0.68**	0.06<br>0.07<br>**0.07**	-0.07<br>-0.17<br>**-0.81**	0.18<br>0.09<br>**-0.55**	community:0.05 ± 0.23	R^2m 0.21<br>R^2c 0.33
**Models of relative yield**									
47	relative yield	R=2	(Intercept)<br>**NPs = 4 mg/L**<br>**NPs = 16 mg/L**	0.83<br>**0.23**<br>**0.56**	0.16<br>**0.11**<br>**0.11**	0.51<br>**0.01**<br>**0.33**	1.15<br>**0.45**<br>**0.78**	community:0.17 ± 0.42<br>species:0.08 ± 0.28	R^2m 0.06<br>R^2c 0.33
48	relative yield	R=4	(Intercept)<br>**NPs = 4 mg/L**<br>**NPs = 16 mg/L**	0.38<br>**0.15**<br>**0.41**	0.08<br>**0.05**<br>**0.05**	0.23<br>**0.06**<br>**0.32**	0.54<br>**0.24**<br>**0.50**	community:0.01 ± 0.11<br>species:0.04 ± 0.19	R^2m 0.10<br>R^2c 0.28
49	relative yield	R=8	(Intercept)<br>**NPs = 4 mg/L**<br>**NPs = 16 mg/L**	0.16<br>**0.12**<br>**0.32**	0.07<br>**0.02**<br>**0.02**	0.02<br>**0.07**<br>**0.27**	0.29<br>**0.16**<br>**0.36**	species:0.04 ± 0.19	R^2m 0.10<br>R^2c 0.33

Table S5 Comparison of the slope of the biodiversity–ecosystem functioning relationships between different nanoparticle concentration treatments.

Contrast	Estimate	SE	df	t-ratio	*P*-value
NPs 4 mg/L – Control	**0.18**	**0.062**	**530**	**2.81**	**0.014 ***
NPs 16 mg/L – Control	**0.41**	**0.062**	**530**	**6.55**	**< 0.001 *****
NPs 16 mg/L – NPs 4 mg/L	**0.23**	**0.062**	**530**	**3.72**	**< 0.001 *****

Table S6 Comparison of the net biodiversity effect (NE), complementarity effect (CE), and selection effect (SE) values between different nanoparticle concentration treatments for different community richness levels.

Index	Contrast	Estimate	SE	df	t-ratio	*P*-value
NE	*For all communities*					
	NPs 4 mg/L – Control	**0.621**	**0.091**	**397**	**6.82**	**< 0.001*******
	NPs 16 mg/L – Control	**1.085**	**0.091**	**397**	**11.93**	**< 0.001*******
	NPs 16 mg/L – NPs 4 mg/L	**0.465**	**0.091**	**397**	**5.11**	**< 0.001*******
	For two-species communities					
	NPs 4 mg/L – Control	**0.481**	**0.197**	**126**	**2.45**	**0.041**
	NPs 16 mg/L – Control	**0.814**	**0.197**	**126**	**4.14**	**< 0.001*******
	NPs 16 mg/L – NPs 4 mg/L	0.333	0.197	126	1.70	0.211
	For four-species communities					
	NPs 4 mg/L – Control	**0.464**	**0.134**	**126**	**3.45**	**0.002**
	NPs 16 mg/L – Control	**0.908**	**0.134**	**126**	**6.76**	**< 0.001*******
	NPs 16 mg/L – NPs 4 mg/L	**0.444**	**0.134**	**126**	**3.31**	**0.003**
	For eight-species communities					
	NPs 4 mg/L – Control	**0.917**	**0.130**	**141**	**7.06**	**< 0.001*******
	NPs 16 mg/L – Control	**1.533**	**0.130**	**141**	**11.80**	**< 0.001*******
	NPs 16 mg/L – NPs 4 mg/L	**0.617**	**0.130**	**141**	**4.75**	**< 0.001*******
CE	*For all communities*					
	NPs 4 mg/L – Control	**0.661**	**0.127**	**397**	**5.21**	**< 0.001*******
	NPs 16 mg/L – Control	**1.766**	**0.127**	**397**	**13.91**	**< 0.001*******
	NPs 16 mg/L – NPs 4 mg/L	**1.105**	**0.127**	**397**	**8.70**	**< 0.001*******
	For two-species communities					
	NPs 4 mg/L – Control	0.461	0.244	126	1.89	0.147
	NPs 16 mg/L – Control	**1.112**	**0.244**	**126**	**4.55**	**< 0.001*******
	NPs 16 mg/L – NPs 4 mg/L	**0.651**	**0.244**	**126**	**2.66**	**0.024**
	For four-species communities					
	NPs 4 mg/L – Control	**0.585**	**0.187**	**126**	**3.13**	**0.006**
	NPs 16 mg/L – Control	**1.647**	**0.187**	**126**	**8.79**	**< 0.001*******
	NPs 16 mg/L – NPs 4 mg/L	**1.062**	**0.187**	**126**	**5.67**	**< 0.001*******
	For eight-species communities					
	NPs 4 mg/L – Control	**0.936**	**0.209**	**141**	**4.48**	**< 0.001*******
	NPs 16 mg/L – Control	**2.537**	**0.209**	**141**	**12.15**	**< 0.001*******
	NPs 16 mg/L – NPs 4 mg/L	**1.601**	**0.209**	**141**	**7.67**	**< 0.001*******
SE	*For all communities*					
	NPs 4 mg/L – Control	-0.040	0.065	397	-0.62	0.810
	NPs 16 mg/L – Control	**-0.680**	**0.065**	**397**	**-10.42**	**< 0.001*******
	NPs 16 mg/L – NPs 4 mg/L	**-0.640**	**0.065**	**397**	**-9.80**	**< 0.001*******
	For two-species communities					
	NPs 4 mg/L – Control	0.020	0.128	126	0.16	0.987
	NPs 16 mg/L – Control	**-0.298**	**0.128**	**126**	**-2.33**	**0.056**
	NPs 16 mg/L – NPs 4 mg/L	**-0.318**	**0.128**	**126**	**-2.48**	**0.038**
	For four-species communities					
	NPs 4 mg/L – Control	-0.122	0.104	126	-1.18	0.469
	NPs 16 mg/L – Control	**-0.739**	**0.104**	**126**	**-7.14**	**< 0.001*******
	NPs 16 mg/L – NPs 4 mg/L	**-0.617**	**0.104**	**126**	**-5.96**	**< 0.001*******
	For eight-species communities					
	NPs 4 mg/L – Control	-0.019	0.097	141	-0.20	0.978
	NPs 16 mg/L – Control	**-1.004**	**0.097**	**141**	**-10.33**	**< 0.001*******
	NPs 16 mg/L – NPs 4 mg/L	**-0.985**	**0.097**	**141**	**-10.13**	**< 0.001*******

Supplementary figures

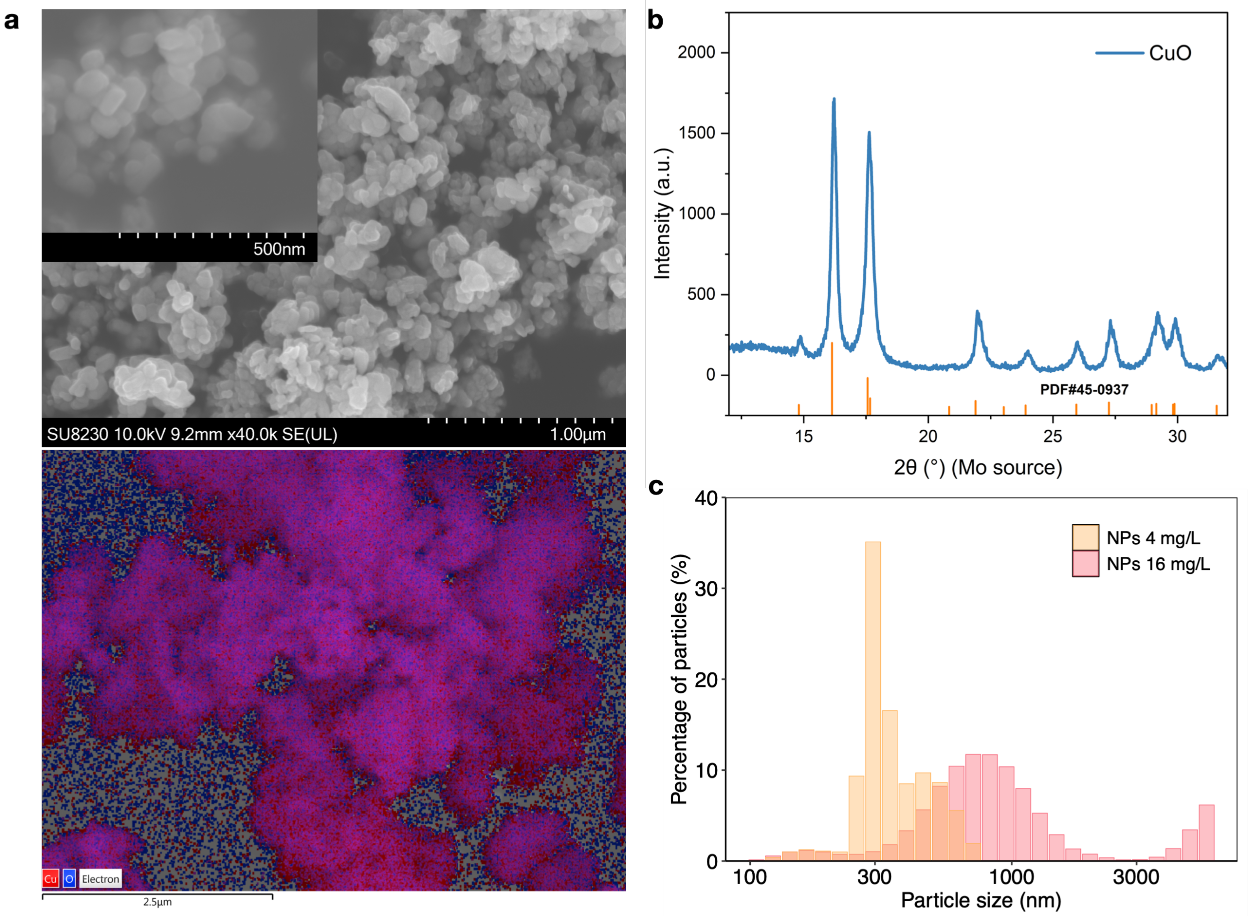

Figure S1. Characterization of CuO nanoparticles. (a) Scanning electron microscopy (SEM) images show the morphology and size distribution of CuO nanoparticles in powdered form, with elemental mapping confirming the distribution of copper (Cu) and oxygen (O). (b) X-ray diffraction (XRD) analysis confirms the phase purity of CuO nanoparticles. (c) Particle size distributions of CuO nanoparticles suspended in the COMBO medium at two tested concentrations (4 and 16 mg/L), measured using a Zetasizer Nano.

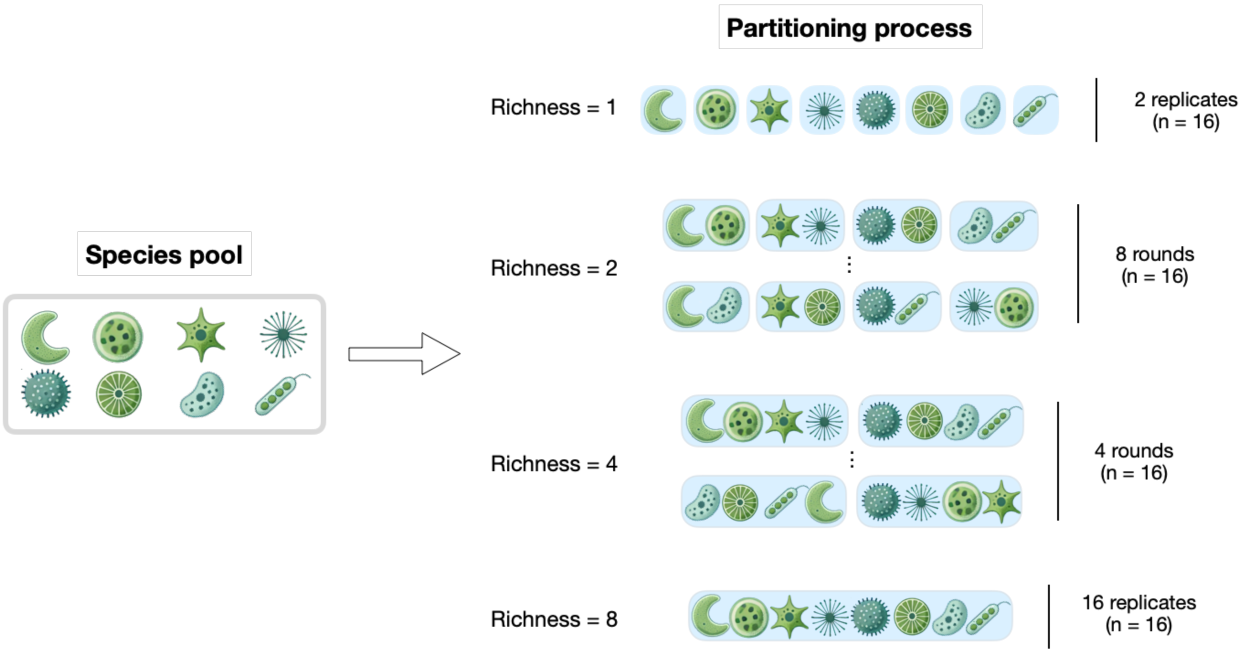

Figure S2. A schematic diagram demonstrating the random partitioning process that we used to select phytoplankton communities of different richness from the eight-species pool.

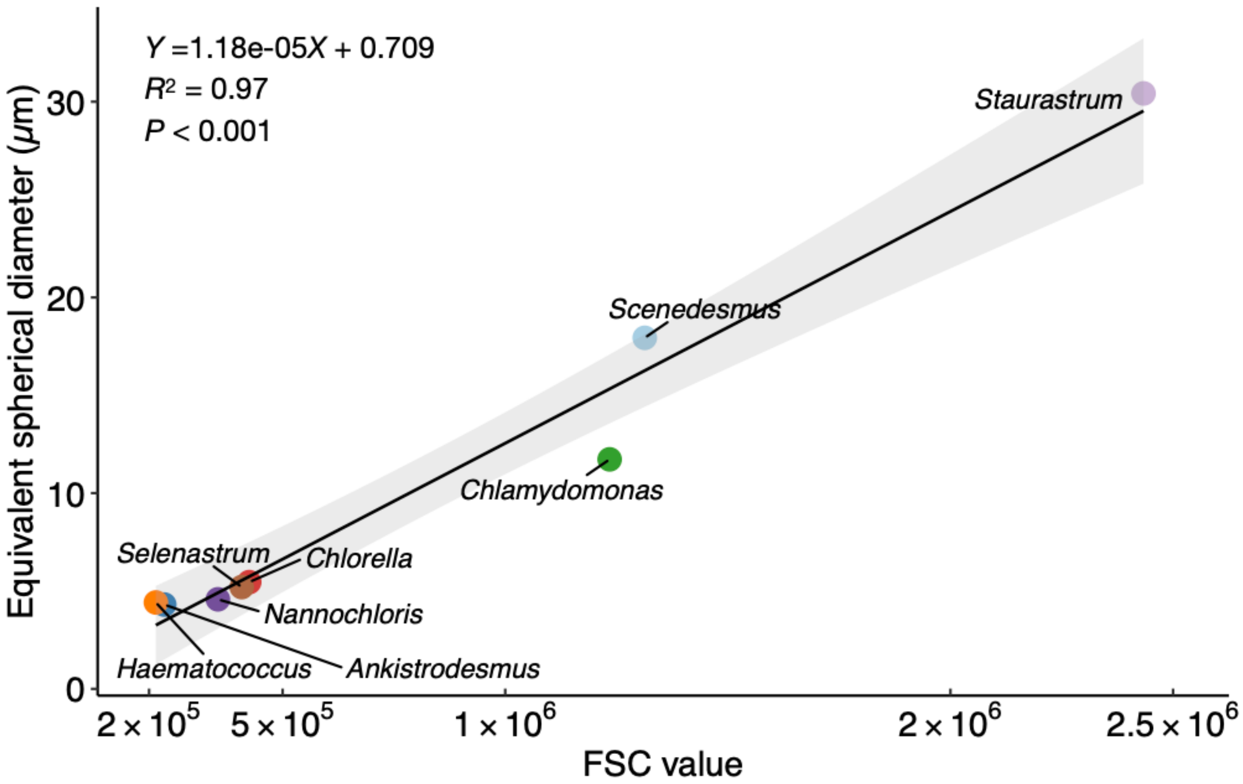

Figure S3. The relationship between forward scatter height (FSC.H) and equivalent spherical diameter (ESD) across the eight phytoplankton species. The regression shows a strong positive linear relationship ($Y = 1.18\text{e–}05X + 0.709$, $R^2 = 0.97$, $P < 0.001$), validating the accuracy of using FSC.H as a proxy for cell size.

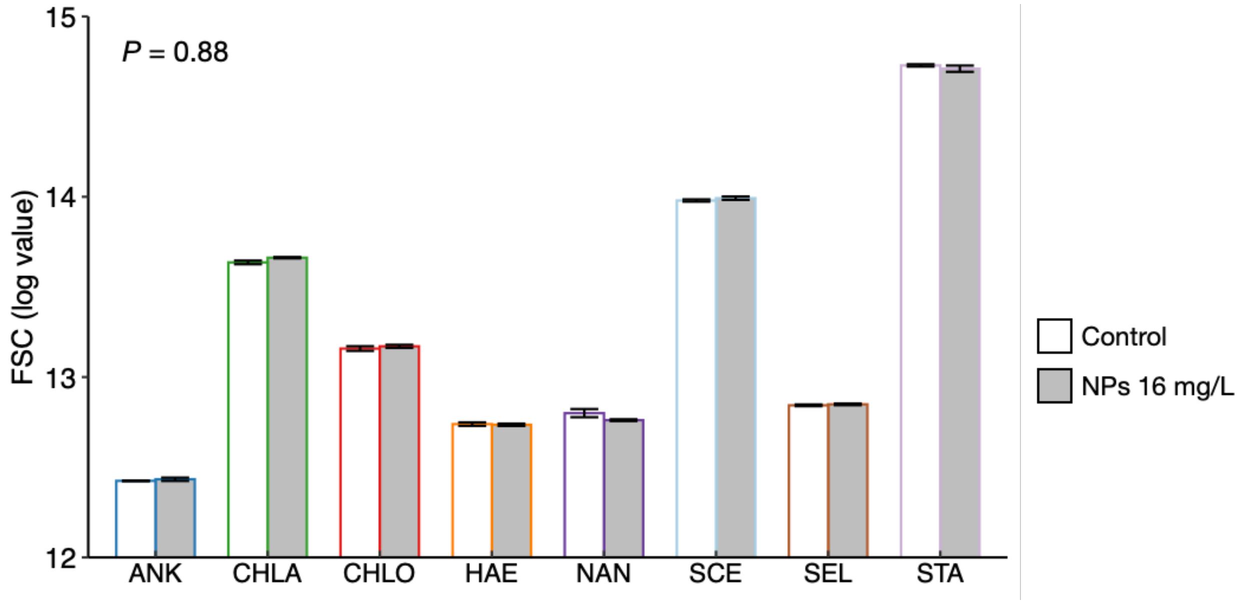

Figure S4. Forward scatter height (FSC.H) values for each phytoplankton species under control and 16 mg/L CuO nanoparticle treatments. Bars show mean log-transformed FSC.H values ± standard errors across three replicates. No significant difference was observed between treatments (P = 0.88), indicating that nanoparticle exposure did not bias FSC.H-based size estimation. Species abbreviations: ANK, *Ankistrodesmus falcatus*; CHLA, *Chlamydomonas sp.*; CHLO, *Chlorella vulgaris*; HAE, *Haematococcus pluvialis*; NAN, *Nannochloris sp.*; SCE, *Scenedesmus quadricauda*; SEL, *Selenastrum capricornutum*; STA, *Staurastrum gracile*.

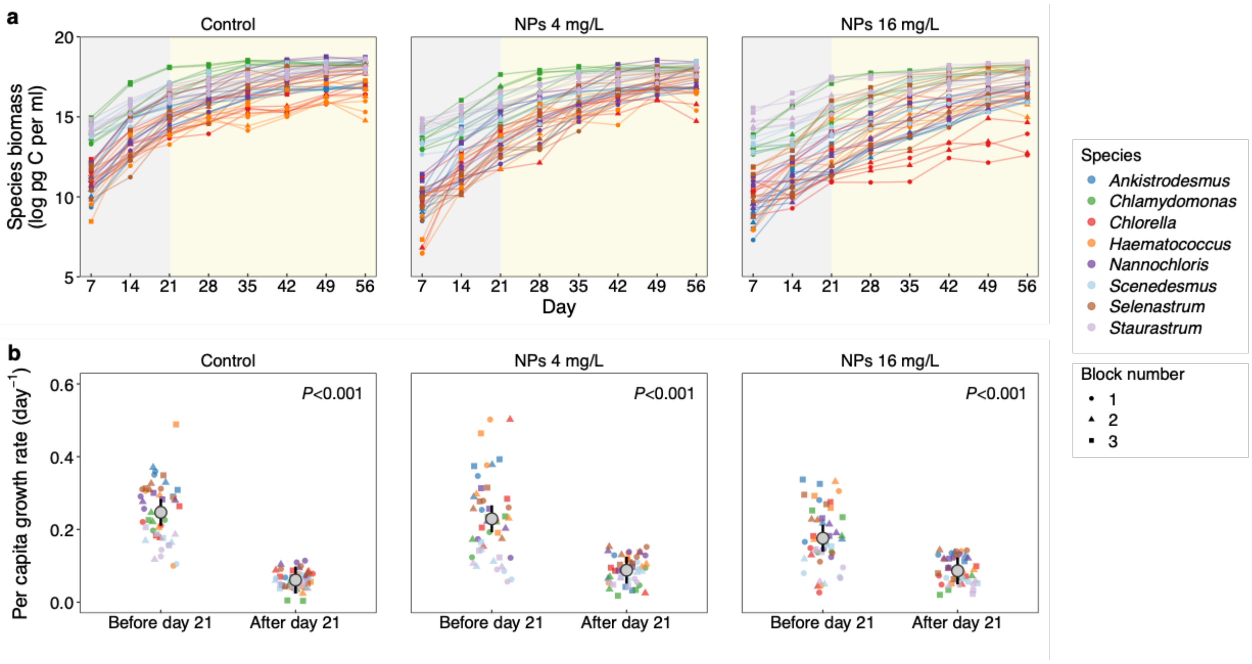

Figure S5. Temporal dynamics of monoculture biomass under different nanoparticle treatments (a) and comparison of growth rates before and after day 21 of our experiment (b). In panel (a), lines represent biomass trajectories of individual species in each microcosm over time. In panel (b), small data points show phase-specific growth rates for individual microcosms, while large points with 95% CI represent average growth rates across all species within a treatment. Growth rates were calculated for each species within each microcosm over seven-day intervals and grouped into two phases (before and after day 21). Linear mixed-effects models, with growth phase as a fixed effect and species as a random effect, indicated consistently significant differences between the two phases across nanoparticle treatments ($P < 0.001$).

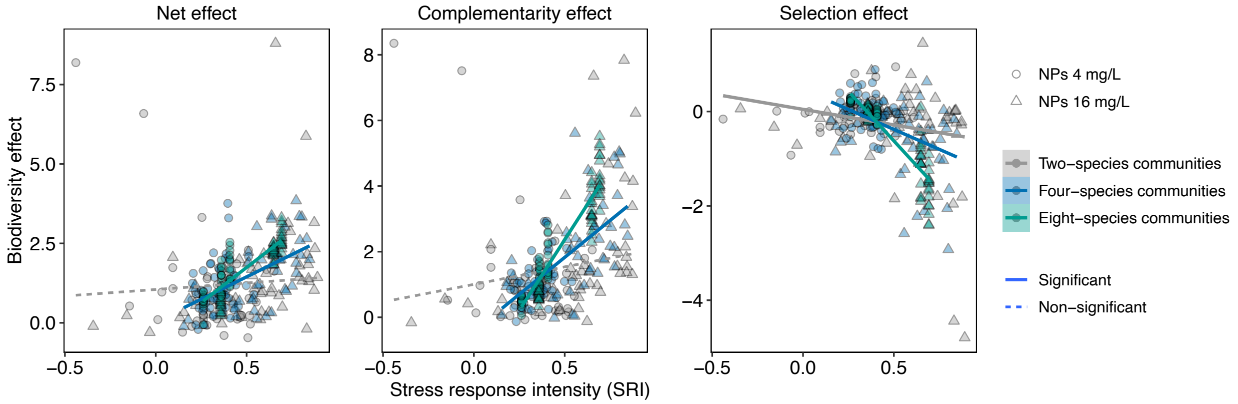

Figure S6. Relationships between biodiversity effects and community stress response intensity (SRI) under nanoparticle exposure. Net biodiversity effect, complementarity effect, and selection effect as functions of SRI across three community richness levels are shown in panels a–c, respectively. Point shapes represent nanoparticle treatments (NPs at 4 mg/L and 16 mg/L), and colors denote community types. Solid lines indicate significant relationships ($P < 0.05$), while dashed lines indicate non-significant trends.

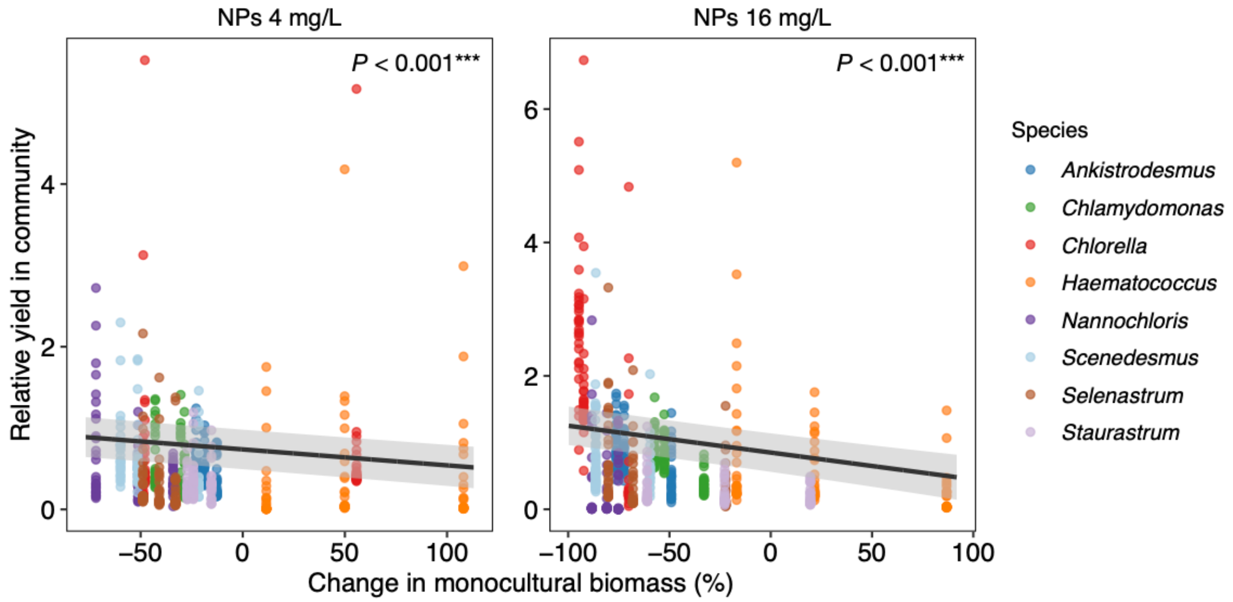

Figure S7. The relationship between the change in monocultural biomass and species relative yield in communities under nanoparticle exposure. Each point represents a species within a specific community at either 4 mg/L or 16 mg/L nanoparticle concentration. The x-axis shows the percentage change in species monocultural biomass under nanoparticle treatment relative to control conditions, while the y-axis shows the relative yield of the same species in multi-species communities.

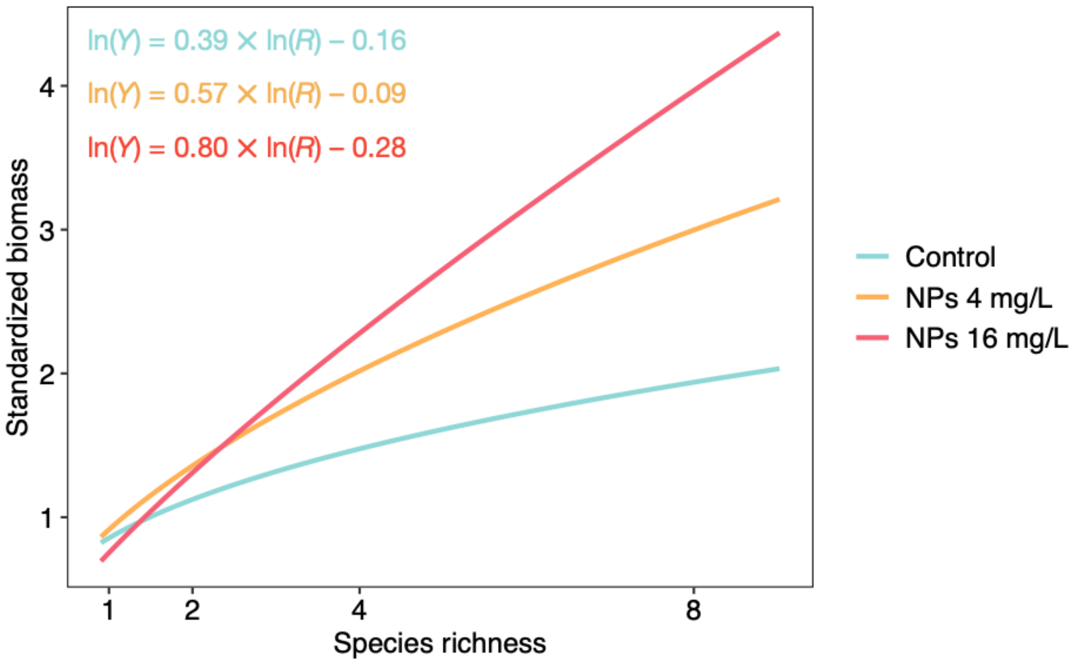

Figure S8. The relationship between community standardized biomass and species richness under different nanoparticle treatments. Standardized biomass was calculated as community biomass divided by the mean monoculture biomass of the constituent species. Log-transformed standardized biomass and log-transformed richness are linearly correlated, with the slope increasing significantly with increasing nanoparticle concentration.

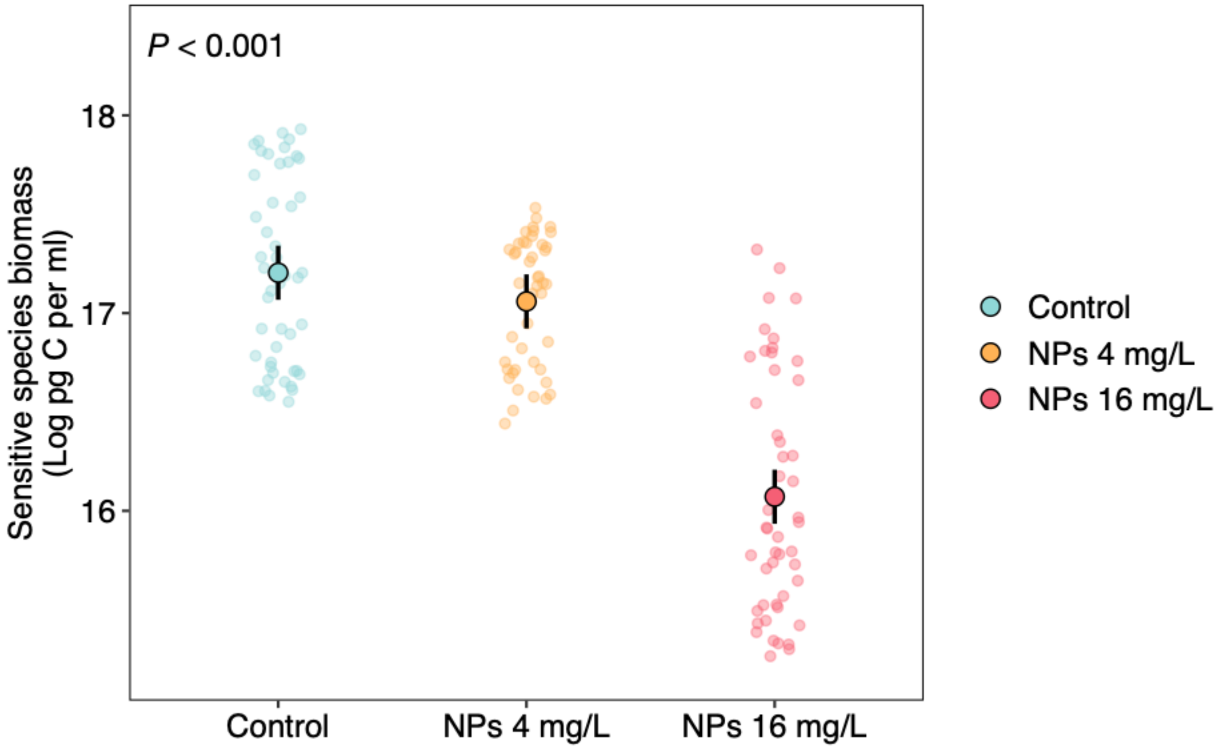

Figure S9. Total biomass of the four most sensitive species (*Chlorella vulgaris*, *Nannochloris sp.*, *Scenedesmus quadricauda*, and *Ankistrodesmus falcatus*) in eight-species communities under increasing nanoparticle concentrations.

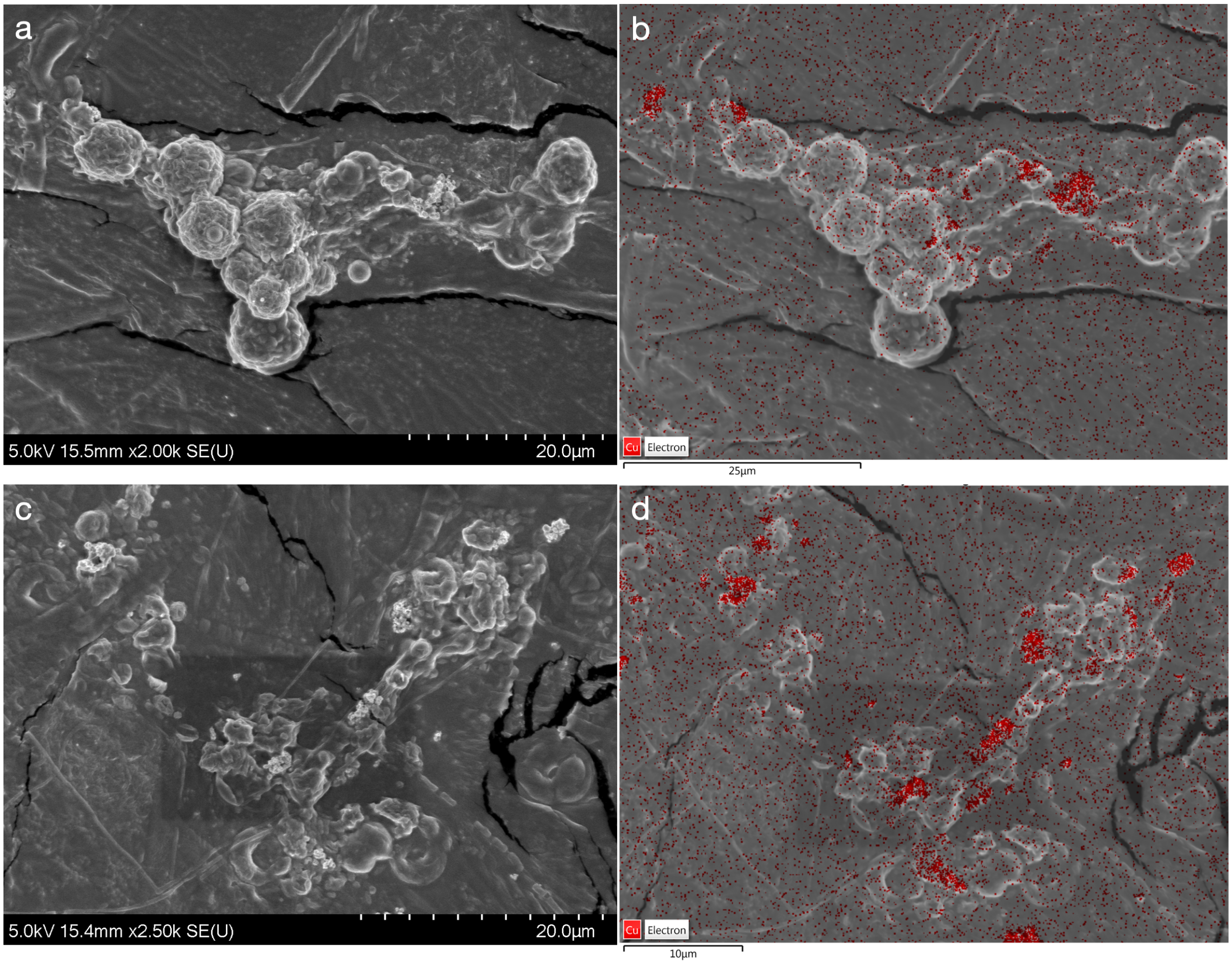

Figure S10. Scanning electron microscopy (SEM) and energy-dispersive X-ray spectroscopy (EDX) analyses showing the attachment of CuO nanoparticles to the surfaces of phytoplankton cells. Panels a and c show two different fields of view under SEM, while panels b and d present the corresponding EDX elemental maps highlighting Cu signals, confirming the adhesion of CuO nanoparticles to the cell surfaces.

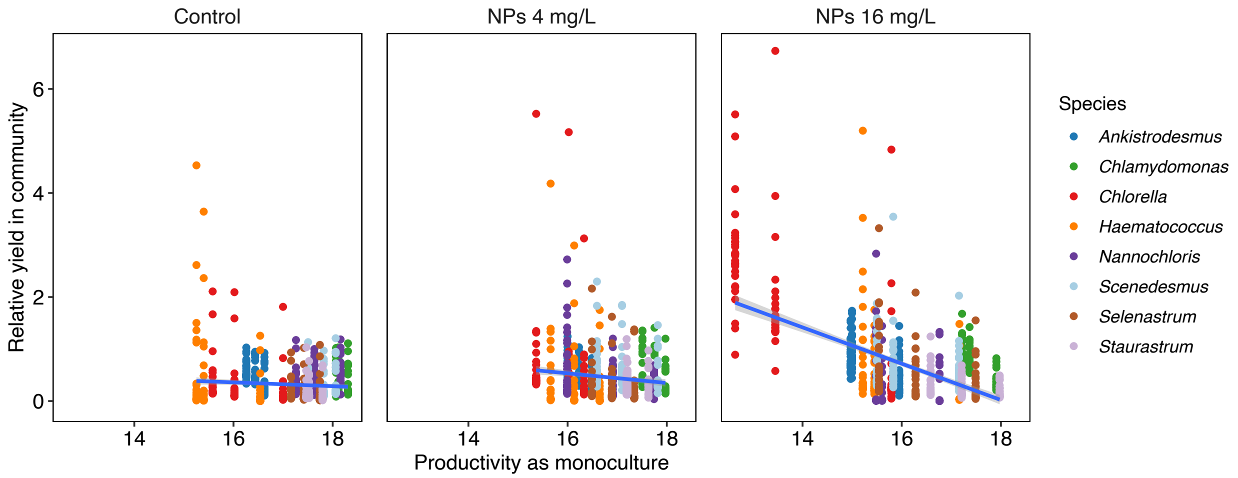

Figure S11. The relationship between species relative yield in multi-species communities and its monoculture biomass under different nanoparticle concentrations.

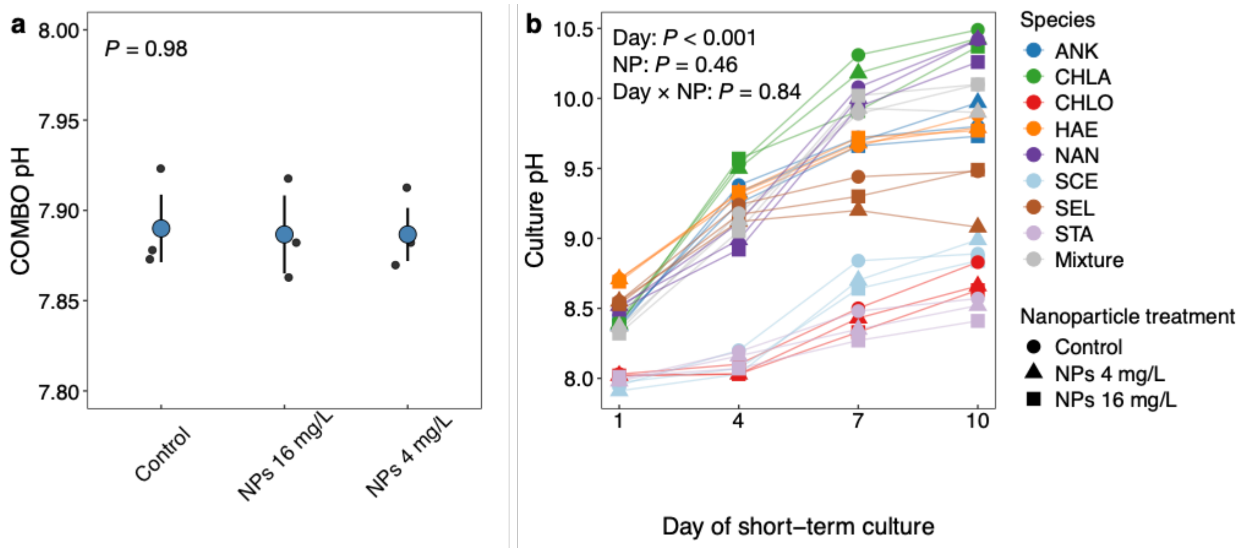

Figure S12. Comparisons of pH in the COMBO medium with the three nanoparticle treatments (a) and temporal changes in the pH of culture medium in a short-term growth experiment (b). In panel a, statistical results were obtained using ANOVA. In panel b, statistical results were obtained using a linear mixed-effects model with day, nanoparticle treatment (NP), and their interaction as fixed effects, and phytoplankton species identity as a random effect. The short-term experiment was conducted independently from the main experiment. pH was measured every three days from day 1 to day 10.
